# Using a Community-Based Early Childhood Development Center as a Platform to Promote Production and Consumption Diversity Increases Children's Dietary Intake and Reduces Stunting in Malawi: A Cluster-Randomized Trial

**DOI:** 10.1093/jn/nxy148

**Published:** 2018-09-10

**Authors:** Aulo Gelli, Amy Margolies, Marco Santacroce, Natalie Roschnik, Aisha Twalibu, Mangani Katundu, Helen Moestue, Harold Alderman, Marie Ruel

**Affiliations:** 1 International Food Policy Research Institute, Washington, DC; 2 Johns Hopkins University, Baltimore, MD; 3 Save the Children, Washinghton/Zomba, USA/Malawi; 4 Chancellor College, University of Malawi, Zomba, Malawi

**Keywords:** early childhood, nutrition, diets, agriculture, impact evaluation

## Abstract

**Background:**

Children in Malawi face nutritional risks related to low-quality diets and chronic malnutrition.

**Objective:**

This study evaluated the impact of a 1-y early childhood development (ECD) center–based agriculture and nutrition intervention aimed at improving household production diversity, maternal knowledge on child nutrition and feeding practices, and children's diets and anthropometric measures.

**Methods:**

A longitudinal cluster-randomized controlled trial was implemented in 60 community-based childcare centers (CBCCs), covering 1248 preschool children (aged 36–72 mo) and 304 younger siblings (aged 6–24 mo). CBCCs were randomly assigned to *1*) a control group providing the Save the Children's ECD program or *2*) a treatment group providing a standard ECD program with additional activities to improve nutritious food production and behavior change communication to improve diets and care practices for young children. Primary outcomes were household production and production diversity, preschooler enrollment and attendance, and dietary intake measured by quantitative 24-h recall and minimum diet diversity for younger siblings. Secondary outcomes included anthropometric measures for preschoolers and younger siblings, child development scores for preschoolers, and women's asset ownership and time use (the latter 2 are not discussed in this article). We used difference-in-difference (DID) estimates to assess impacts.

**Results:**

Compared with the control group, preschool children in the intervention group had greater increases in nutrient intakes and in dietary diversity. No impacts on anthropometric measures were seen in preschoolers. Younger siblings in the intervention group had greater increases in height-for-age *z* scores than did children in the control group (DID: 0.44; *P* < 0.05) and greater reductions in the prevalence of stunting (DID: –17 percentage points; *P* < 0.05). The plausibility of the impact on growth in younger siblings was supported by effects along program impact pathways, including production of nutritious foods, caregiver knowledge, and dietary diversity.

**Conclusion:**

Implementing an integrated agriculture and nutrition intervention through an ECD platform benefited children's diets and reduced stunting among younger siblings of targeted preschoolers. This trial was registered on the ISRCTN registry as ISCRCTN96497560.

## Introduction

Estimates of the global burden of malnutrition indicate that undernutrition causes >3 million child deaths/y (1) and that 155 million children aged <5 y are stunted (2). Deficiencies in micronutrients also contribute to increased child and maternal mortality while impairing children's physical and mental development (3).

Reviews of the contributions of nutrition-sensitive development, and agricultural programs in particular, conclude that although such programs have the potential to improve nutrition, this potential is yet to be fully realized (4, 5). Limitations in the design and implementation of nutrition-sensitive agricultural interventions, as well as the lack of rigor in impact evaluations, prevent clear conclusions with regard to their contribution in improving nutrition (5). More-recent evidence suggests that well-designed and carefully implemented nutrition-sensitive agricultural programs improve maternal and child nutrition and are effective at increasing intakes of nutritious foods and improving diet quality when they include a strong behavior change communication (BCC) and women's empowerment interventions (6). Early childhood development (ECD) programs are another platform recommended for delivering nutrition interventions to preschool children (5). One justification for using ECD platforms to deliver nutrition interventions is the potential for synergies between ECD and nutrition on both child development and nutrition outcomes (7, 8). Another justification is that, with the recognition of the importance of the period from conception to the child's second birthday, the focus of nutrition programs has shifted to this period, and as a result, children aged 2–6 y (preschoolers) are left out of many nutrition and health programs until they enroll in school. Although preschool children may have less potential to benefit from nutrition interventions in terms of linear growth, they still have nutritional needs, including receiving a nutritious and healthy diet that allows them to meet their nutrient requirements. Integrated ECD and nutrition investments can thus provide a way to maintain a continuum in nutrition programming among children beyond the first 2 y. Moreover, ECD programs can be leveraged to reach caregivers and promote healthy diets among all other household members, including younger siblings.

The national ECD program in Malawi is led by the Ministry of Gender, Children, and Social Welfare and consists of support to preschools [known as community-based childcare centers (CBCCs)] and parenting groups. CBCCs are community-led centers that promote child development by providing safe and stimulating environments, access to health and nutrition services, and training for parents and caregivers. CBCCs service children aged 3–6 y and are open from 0800 to 1100, 5 d/wk. When possible, a porridge is provided midmorning with food contributions from the community. However, an irregular supply of food has been reported as one of the main causes of child absenteeism and CBCC closure (9).

The Nutrition Embedded Evaluation Program Impact Evaluation (NEEP-IE) used a cluster-randomized controlled trial (CRCT) design to examine the effectiveness of using a community-based ECD center as a platform to promote household production and consumption diversity, improve caregiver knowledge and practices of nutrition and infant and young child feeding (IYCF) practices, and improve diets and nutrition among preschoolers and their younger siblings (10). This study presents the impacts on all of the primary outcomes of the trial and the nutrition-related secondary outcomes (anthropometrics). Impacts on the other secondary outcomes, including preschoolers’ cognitive development and women's asset ownership, time use, and productivity were also assessed and will be reported in separate publications.

By testing the effectiveness of the intervention through community-based ECD centers, this study addresses an evidence-base gap on innovative delivery platforms for nutrition-sensitive interventions (11). Although the primary target group in this study was preschoolers, the intervention involved their parents and caregivers as entry points to influence household decisions and potentially reach younger siblings during a critical period for their growth. Through the analysis of intermediate outcomes along theorized program impact pathways, the study aimed to establish plausibility of findings and identify the channels through which impacts may have been achieved (12).

## Methods

### Country context

Malawi has one of the highest rates of chronic malnutrition in the world, with 37% of children aged 6–59 mo being moderately or severely stunted (13). Severe climatic shocks and flooding in 2014–2016 resulted in high levels of food insecurity across the country, leaving 2.8 million people in need of humanitarian support (14, 15). The situation worsened the following year with widespread drought (16). After several years of agriculture-led growth and consistent improvements in health and nutrition indicators (17), these shocks reversed momentum and risked serious long-term negative effects on the population's health and nutrition.

### Intervention description

#### The standard package

Save the Children (SC) has supported CBCCs in the Zomba district of Malawi since 2008. The standard ECD package provided to SC-supported communities is based on materials developed by the Government of Malawi. As part of this study, caregivers in all the CBCCs received a 2-wk training provided by government-approved trainers who conducted counseling sessions using a government manual. The topics discussed included child nutrition and stimulation and parental role in school readiness. Caregiver groups were led by the trained facilitators 1 time/mo for the study duration. As part of the training, links between CBCCs and parenting groups were strengthened with the aim of improving parenting practices and also reaching younger siblings.

#### The integrated intervention

The NEEP-IE integrated agriculture and nutrition intervention aimed at increasing the effectiveness of the government ECD program. The agriculture component promoted improved production of nutritious foods and food diversification by using CBCC gardens as a demonstration site for communities. Before the 2 main planting seasons, government agriculture extension development officers (AEDOs) held 3 d of training for parents, CBCC Management Committee representatives, farmers, and community agents on land preparation, selection of nutritious crops, agriculture production techniques, pest and disease management, manure-making and application, harvesting, storage, processing, and chicken rearing. Village savings and loans groups were also supported by SC to start home gardens and help communities purchase supplies for CBCC meals. The agriculture training focused on nutritious food production, including a traditional variety of orange maize (rich in vitamin A) and biofortified orange-fleshed sweet potato, legumes and nuts (soya beans, pigeon peas, cowpeas, and groundnuts), and green leafy vegetables (amaranthus), as well as care for chickens. Participating households received seeds along with 10 chicks.

The nutrition component was aimed at improving feeding and caring practices and engaging parents and other caregivers in the planning and preparation of meals in CBCCs. Activities included BCC and training in nutritional needs of infants and young children, year-round meal planning and preparation, food storage, hygiene, waste disposal, and monitoring of meal provision. Recipes included preparation of nutrient-rich meals based on seasonal foods. CBCC Management Committee members, CBCC caregivers (teachers), lead farmers, and parents received a 3-d nutrition training session by government AEDOs and nutrition assistants. By taking turns preparing CBCC meals throughout the study period, parents continued to practice new recipes at the CBCC, which they then replicated at home. The first set of agriculture training sessions was implemented after the baseline survey in December 2015, before the planting season. The nutrition training began in February 2016. Monthly follow-up visits were undertaken by AEDOs and SC staff.

### Program theory

**FIGURE 1:**
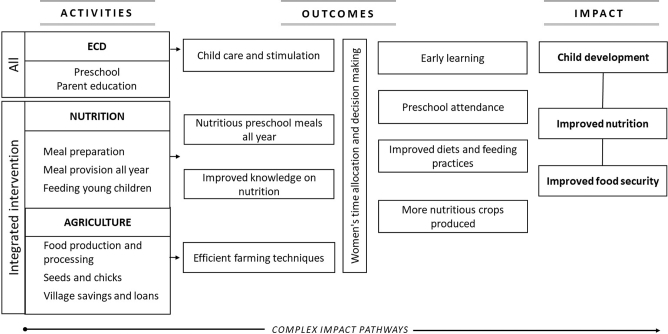
Schematic view of the program impact pathways for the integrated agriculture and nutrition intervention in the NEEP-IE study. NEEP-IE, Nutrition Embedded Evaluation Program Impact Evaluation.

The program theory for the integrated agriculture and nutrition intervention was guided by the Lancet Series framework on Maternal and Child Nutrition (1) and the framework describing pathways by which agriculture can improve nutrition (18) through 3 channels (**Figure 1**). First, the intervention could affect agriculture by increasing production, improving the household-level availability of nutritious foods. Second, the nutrition BCC could improve diets and feeding practices by improving caregiver knowledge. And third, by increasing the regularity and quality of CBCC meals, the intervention could influence CBCC participation, possibly enhancing both their learning and nutritional status (19).

### Study design and participants

**FIGURE 2:**
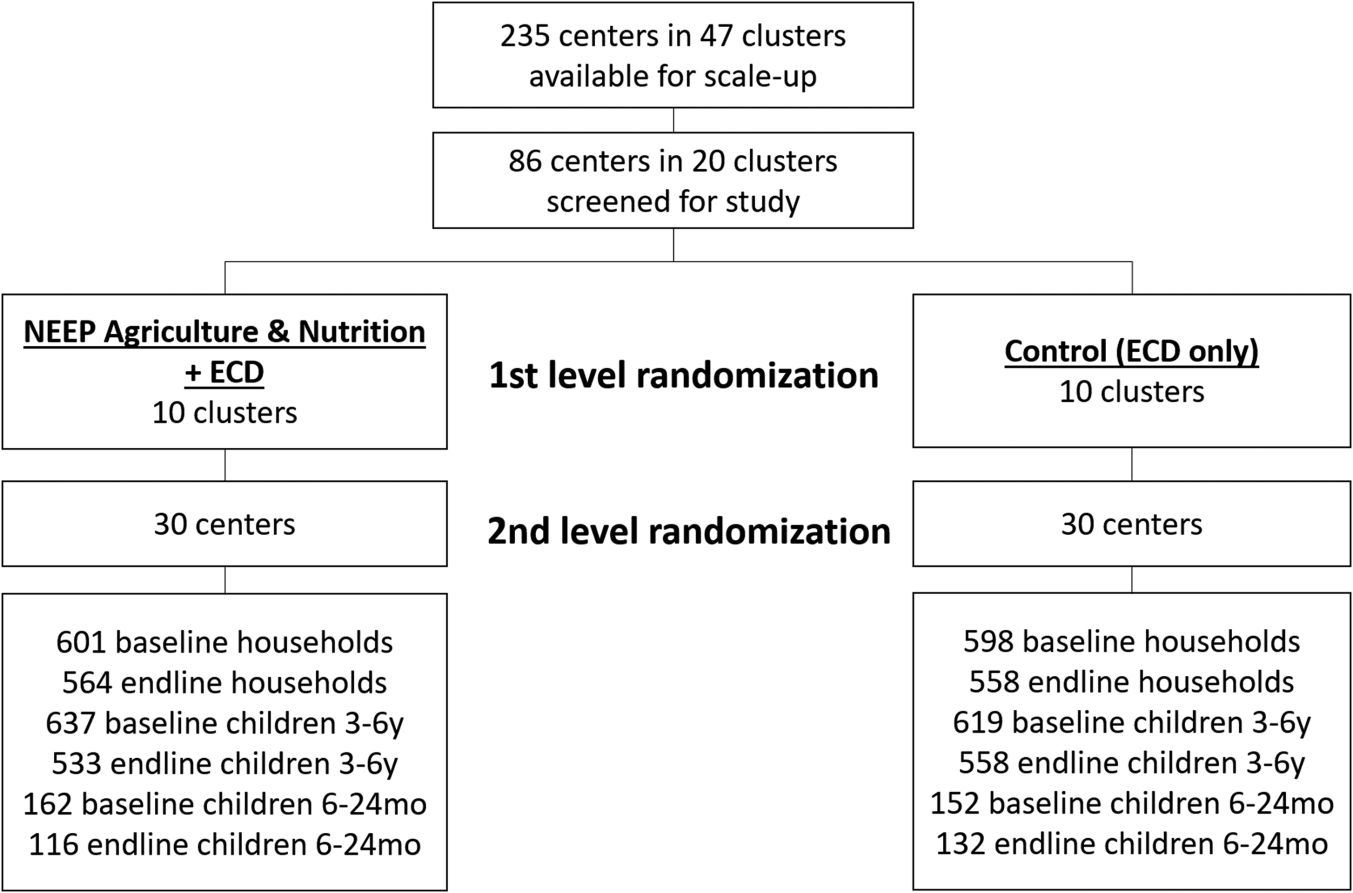
Schematic view of the randomization process and trial profile. ECD, Early Childhood Development; NEEP, Nutrition Embedded Evaluation Program.

A CRCT was implemented in 60 rural communities with CBCCs supported by the SC program in the Zomba district, Malawi. The CRCT study protocol is published elsewhere (10). The evaluation combined quantitative and qualitative methods with 2 rounds of surveys timed 12 mo apart. Communities were randomly assigned to 1 of 2 arms (**Figure 2**), as follows: *1*) a control group (communities with CBCCs supported by the SC ECD program) and *2*) an intervention group (communities with CBCCs supported by the SC ECD program with an additional agriculture and nutrition intervention).

The intervention was implemented in 30 of the 60 rural communities after the baseline survey. Several reasons explain why the control group in this case was not a control without an intervention. The government of Malawi is committed to scaling up the ECD support activities across all CBCCs and an impact evaluation on the cost-effectiveness of different ECD strategies is underway. This study complemented ongoing work by examining the relative impact of alternative models, focusing on how to enhance participation in the CBCCs and support nutrition of children at a critical age in their development.

The 60 CBCCs were randomly selected in 2 stages from a pool of 235 CBCCs located in 47 primary school clusters assisted by SC. Due to the clustering of the CBCCs around primary schools, the list of 235 CBCCs was screened to flag clusters where >1 CBCC was supported. The cluster (unit of randomization) was the primary school cluster that included several CBCCs. Twenty-seven clusters with ongoing training activities in >1 CBCC were excluded from the first stage of randomization to minimize possible contamination. Twenty clusters were then randomly assigned to 2 groups of 10 clusters, where randomization was stratified geographically across 3 traditional authority areas. In the second stage of randomization, 3 CBCCs were selected at random within each cluster. Because 6 clusters had <3 CBCCs, a larger number of CBCCs was randomly selected from 3 other clusters to allow selection of a full sample of 30 CBCCs/arm. The number of CBCCs/cluster ranged from 1 to 6 (mean = 3.5 and median = 3). The random allocation was undertaken using the “sample” command in STATA with a random seed set to the serial number of the first currency bill drawn from the first author's wallet. Enumerators were not blinded to the allocation.

The study targeted all children aged 6–72 mo and their caregivers in the 60 communities. The primary reference group included children aged 36–72 mo at baseline (preschooler group) living in the service area of SC‐supported CBCCs. A secondary reference group included all children aged 6–24 mo at baseline (younger sibling group) living in households with ≥1 other child in the preschooler group. Primary outcomes included household food production and production diversity, individual dietary intake and dietary diversity score (DDS; in preschoolers), CBCC enrollment and attendance (in preschoolers), and DDS and minimum dietary diversity (MDD; in younger siblings). Secondary outcomes included anthropometric measures (weight-for-age, height-for-age, and weight-for-height *z* scores; WAZ, HAZ, and WHZ, respectively) for all children aged 6–72 mo; child development in preschoolers (not reported in this article); and women's asset ownership, time use, and productivity (not reported in this articles). The scope of this study is limited to the analysis and reporting of all of the primary study outcomes and the single secondary outcome (anthropometric measures) per protocol that are most relevant to nutrition. The 2 remaining secondary outcomes, including child development and women's asset ownership, time use, and productivity, will be the focus of a set of similar analyses that will be published separately in thematic journals.

### Sample sizes

On the basis of initial power calculations and resource availability, we originally planned for 30 clusters (CBCCs)/treatment arm, with 20 households in each cluster to identify reasonable treatment impacts of the intervention on the primary outcomes. However, after preliminary community visits before the baseline survey, the original sampling strategy was modified to account for the implementation approach adopted by SC involving the clustering of CBCCs around primary schools. Adjusting for intracluster correlation coefficients at the primary school cluster level, where 60 CBCCs were clustered into 2 groups of 10 primary school clusters with 3 CBCCs each, would provide 80% power to detect a 0.4-SD difference in the individual DDS between treatment groups at the 5% significance level. The sampling of households was conducted through a census within a catchment area for each CBCC. Households with children in the preschool reference age group were then randomly selected for study participation.

### Data collection

The baseline and endline surveys were completed in December 2015 and December 2016, respectively. Anthropometric measurements were also collected at midline in April 2016.

#### Household food production

Agricultural production was estimated for each crop cultivated in the previous 12 mo during the household interview. A production diversity index was calculated as a count of the number of food groups produced during the previous 12 mo (12 food groups were included; the scale scores ranged from 0 to 12) that was identical to that used to measure dietary diversity (20, 21). A production variety index was computed as the total count of the number of individual crops that households reported cultivating during that same period.

#### Caregiver IYCF knowledge and practices

Knowledge of IYCF was assessed through caregiver recall in 2 ways. We first elicited knowledge by asking caregivers open-ended questions on what they knew about the nutritional needs of infants (aged 0–6 mo) and young children (separately for 6- to 24-mo and 2- to 5-y age groups; e.g., “What do you know about the needs of children aged 0–6 mo regarding feeding?”). We subsequently asked caregivers a battery of questions on knowledge of specific practices (e.g., “How long after birth should a baby start breastfeeding?”) as described in WHO guidelines (22). Caregivers were also asked about their knowledge of food groups and of the properties of foods within different food groups. Open-ended question responses were coded and analyzed both individually and by generating a food-group knowledge score that aggregated responses of relevant questions (with scores ranging from a minimum of 0 to a maximum of 6).

#### Children's diets, IYCF, and anthropometric assessment

Dietary assessment was undertaken using the interactive multi-pass 24-h recall method (23). To estimate the distribution of usual intake and account for within-person variation (24), a subset of 120 households was selected to have two 24-h recalls ≥2 d apart. Before the recall interview, caregivers were briefed on the purpose and methods of interview. Interviews were conducted with the use of visual aids to assist in estimating portion sizes. Individual recipes were broken down into individual ingredients at the household level. A preferred method was established for each food, including direct weighing, standard portion sizes, and calibrated portion-size models. Quantities in grams of different food items that children had consumed over the past 24 h were converted into nutrients with the use of a food-composition table adapted for Malawi (25), adjusting for nutrient retention factors of cooked foods (26). Outliers in caloric intake with values >3 SDs were excluded from the nutrient intake analysis. The assessment did not include meals provided in CBCCs, because caregivers at home were in most cases not aware of specific details of meals provided in CBCCs or actual quantities consumed by children. Meal provision data were collected at CBCCs, including recipes, ingredients, and quantities provided per child, although these were not included in the child-level estimates of food intake. Child dietary diversity (for preschoolers) and household dietary diversity were measured with the use of the DDS, calculated as a count of the number of food groups consumed by children in the 24-h assessment (20, 21) and in a household food-consumption 7-d recall (27). Twelve food groups were included (the scale of the scores was 0–12). It is important to note that no dietary diversity indicator has been validated for preschool children; current validated dietary diversity indicators exist only for children aged 6–24 mo (22) and for women (28). At the household level, we also used the household food variety score, calculated as a count of the number of individual food items that households reported consuming in the previous week (21).

At endline, we included the WHO MDD indicator for younger siblings (22). The MDD score was calculated as the prevalence of children consuming a count of ≥4 of 7 food groups during the previous 24 h as per WHO guidelines. Anthropometric data included measurements of height and weight for all children from 6 to 72 mo old undertaken during home visits. Recumbent length of children aged <2 y and standing height of children aged >2 y was measured to the nearest 0.1 cm by using portable fixed-base stadiometers or length boards; weight was measured to the nearest 0.1 kg with the use of electronic scales. All enumerators collecting anthropometric data were trained using standard WHO guidelines, and measurements (29) were practiced before the survey through standardization exercises. From these standardization sessions, inter- and intraobserver variations in measurement error were documented and the necessary corrections to procedures were made. All of the measurements were undertaken by an anthropometrist and an assistant. Linear growth was examined by using the HAZ and prevalence of stunting. HAZ, WAZ, and WHZ scores were calculated by using the 2006 WHO growth standard using WHO cutoffs (29). Stunting was defined as HAZ <−2 SDs, wasting as WHZ <−2 SDs, and underweight as WAZ <2 SDs. RDAs were obtained from references 30 and 31. All data (including dietary assessments) were collected by trained enumerators using electronic, android-based tablets with computer-assisted personal interview software. An additional back-check survey for quality-assurance purposes was conducted in a random sample of 120 households. Ethical clearance was obtained from ethics boards at Chancellor College, the University of Malawi (reference: NCST/RTT/2/6), and the International Food Policy Research Institute. Informed consent was obtained from parents through written and verbal information provided before interviews.

### Statistical analysis

The analysis followed an intention-to-treat approach as per the published protocol (10). The impact on dietary intake in preschool children and anthropometric measures in children aged 6–72 mo was assessed with a difference-in-difference (DID) estimator by using multilevel regression models accounting for the hierarchical nature of the data (32). The multilevel models used fixed effects and random effects at cluster and household levels. The DID estimate was calculated as the average change in the outcome of interest between baseline and endline in the intervention arm minus the change in outcome in the control arm. The impact on the dietary diversity IYCF indicator in younger siblings was estimated by using single differences at endline because no baseline information was available. Regression models for child-level indicators were adjusted for sex and age. The regressions used linear probability models for both continuous and binary variables for ease of interpretation unless otherwise specified. Impacts were considered significant at *P* < 0.05. Robustness analysis for the anthropometry impact estimates in younger siblings included data from the midline survey. The robustness analysis also included comparing regression results for younger siblings at each time (“full sample”; *n* = 304) with those measured at all 3 time points only (“full cohort”; *n* = 208). The study was registered on the ISCRCTN registry (ISCRCTN96497560). Because the allocation of clusters to study arms was random, according to procedures in reference 33, significance tests of differences at baseline were not undertaken.

## Results

### Trial attrition

A total of 1199 households and 60 CBCCs in the Zomba district were surveyed at baseline. The endline survey included 1122 households in 60 CBCCs, leading to a 7% attrition rate at the household level. The main reason for attrition included households that moved out of the study area (64 households); other reasons included deaths (4 children) and refusals for re-interviews. The attrition rate was not significantly different between treatment groups nor was the probability of attrition correlated with treatment assignment. No significant differences in means of dietary intake outcomes or HAZ between attrited and nonattrited children who were and were not lost to follow-up (**Supplemental Table 1**).

### Baseline characteristics and tests of balance

At baseline, the average household size was 5.3 members and close to 1 in 3 households were headed by a woman. Thirty-five percent of household heads had completed primary education. Among mothers, only ∼20% had completed primary education. The prevalence of child stunting was high (∼40%), whereas wasting was almost nonexistent (1–3%), similar to the country-level status reported in the latest Demographic and Health Survey (DHS) (13). On average, 26% of households had children in both preschool and younger sibling age groups, a rate similar to that in the latest DHS (27%). Examination of the age distribution in younger siblings at baseline indicated that approximately half were aged between 6 and 12 mo and half were aged between 12 and 24 mo. CBCC participation was high, with >90% of preschool children enrolled in a CBCC; and attendance rates were nearly 80% in the 5 d before the survey. However, only 26% of children reported receiving meals in CBCCs, and meals were provided, on average, for only 1 d out of 5, highlighting a role for the intervention in increasing the regularity of meal provision. Overall, no substantive differences between intervention and control groups were found in the baseline characteristics of the study population (**Table 1**).

**TABLE 1:** Characteristics of the study population at baseline in treatment and control communities: Zomba district, Malawi^1^

	Treatment		Control	
Variable	Value	*n*	Value	*n*
Household				
Household size, *n*	5.32 ± 1.92	601	5.35 ± 1.68	598
Children, *n*				
0–36 mo	0.50 ± 0.58	601	0.52 ± 0.58	598
>36–72 mo	1.13 ± 0.37	601	1.12 ± 0.35	598
>6–14 y	1.44 ± 1.21	601	1.47 ± 1.13	598
Adults, *n*				
>14–65 y	2.25 ± 1.00	601	2.26 ± 0.97	598
>65 y	0.05 ± 0.24	601	0.04 ± 0.22	598
Dependency ratio	1.56 ± 0.96	601	1.6 ± 1.03	598
Household head completed primary school, %	32	601	38	598
Household head's age, y	36.8 ± 10.07	601	36.2 ± 10.3	598
Polygamous households, %	2	601	4	598
Female-headed household, %	27	601	29	598
* *Asset ownership,^2^*n*				
Large livestock	0.02 ± 0.24	601	0.05 ± 0.56	598
Small livestock	0.87 ± 2.86	601	0.73 ± 1.9	598
Fowl (chickens)	2.85 ± 5.42	601	2.30 ± 5.07	598
Farm equipment	3.18 ± 2.57	601	3.07 ± 2.91	598
Small consumer durables	14.49 ± 40.01	601	13.51 ± 14.8	598
Total asset count	23.57 ± 42.42	601	21.34 ± 18.9	598
* *Expenditures, MWK/d per capita^3^				
Total	252 ± 202	576	232 ± 172	563
Nonfood	61 ± 86	576	52 ± 66	563
Food	191 ± 157	576	181 ± 143	563
Mother				
Completed primary school, %	19	962	21	956
Age, y	29.5 ± 7.51	859	29.9 ± 7.33	887
Children				
Girls, %	50	962	52	956
Stunting, 6–24 mo, %	41	155	41	149
Wasting, 6–24 mo, %	1	158	3	150
Underweight, 6–24 mo, %	14	157	13	150
Stunting, 36–72 mo, %	40	615	39	601
Wasting, 36–72 mo, %	1	494	2	465
Underweight, 36–72 mo, %	17	517	17	494
CBCC enrollment, %	92	656	93	645
CBCC attendance, last 5 d, %	81 ± 27	576	77 ± 30	552
Days CBCC open, last 5 d, *n*	4.23 ± 1.42	606	4.38 ± 1.41	600
Received meals, last 5 d, *n*	0.24 ± 0.43	656	0.29 ± 0.45	645

^1^All unadjusted baseline and endline values are means ± SDs unless otherwise indicated. CBCC, community-based childcare center; MWK, Malawian kwacha.

^2^Asset count included 13 asset-type categories where respondents indicated ownership and number of assets owned.

^3^Excludes outliers for food consumption and total expenditure.

### Impact on household food production and consumption

At the household level, positive effects were observed on production diversity and on production of nutritious foods (biofortified orange-fleshed sweet potato, groundnuts, pigeon peas, and soya), as well on chickens owned and eggs produced in the 3 mo before the survey (**Table 2**). Overall, production of these commodities was low at baseline, but significant increases were seen for products targeted by the intervention. No effects were found on household expenditures (including consumption from own production), suggesting that the intervention did not act as an income transfer. Positive effects were found on household dietary diversity (data not shown), suggesting that the intervention resulted in households consuming different foods.

**TABLE 2:** Unadjusted mean household crop diversity and production of nutritious foods at baseline and after 12 mo in the intervention and control groups and adjusted DID impact estimates in households living in treatment and control communities in Zomba district, Malawi: NEEP-IE study^1^

	Treatment (*n* = 542)		Control (*n* = 580)		DID	
Indicator	Baseline	Endline	Baseline	Endline	Impact	SE
Crop production diversity score	3.52	3.52	3.53	2.82	0.71***	0.10
Crop production variety score	6.62	7.87	6.54	5.67	2.14***	0.35
Production of OFSP, kg	1.55	5.62	1.47	1.05	4.32***	0.73
Production of brown beans, kg	3.71	0.70	2.86	0.70	−0.90**	0.44
Production of pigeon peas, kg	14.40	22.84	17.53	21.55	4.86**	1.87
Production of groundnuts, kg	6.59	9.27	7.31	6.68	3.38**	1.63
Production of soya beans, kg	0.37	1.73	0.16	0.08	1.45***	0.14
Chickens owned, *n*	2.84	3.40	2.30	1.71	1.16**	0.45
Egg production past 3 mo, *n*	4.44	5.46	3.83	1.23	3.44**	1.21

^1^All unadjusted baseline and endline values are means. **P < 0.05, ***P < 0.001. DID, difference-in-difference; NEEP-IE, Nutrition Embedded Evaluation Program Impact Evaluation; OFSP, orange-fleshed sweet potato.

### Impact on caregiver knowledge

Positive effects were found when eliciting responses from open-ended questions on broad nutrition topics related to IYCF practices (**Supplemental Table 2**). However, when participants were asked questions on specific knowledge of IYCF practices, no effects of the intervention were found.

Positive impacts were also found on caregiver knowledge related to the importance of different food groups (**Supplemental Table 3**). These effects were driven by knowledge of foods considered important for growth (including beans and groundnuts) and foods needed for energy (including fats and oils). Further analysis of the sources of caregiver knowledge highlighted that SC (the implementer of the intervention) was the main knowledge source for messages on feeding practices (**Supplemental Table 4**).

### Impact on CBCC meals and participation

**FIGURE 3:**
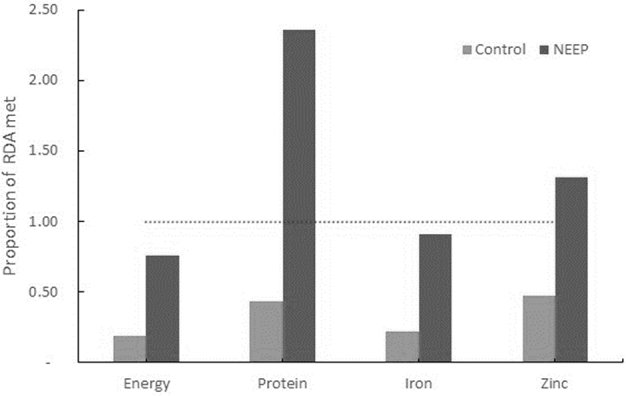
Proportion of RDA provided by meals in CBCCs (*n* = 25 CBCCs) the day before the survey, Zomba district, Malawi: the NEEP-IE study. CBCC, community-based childcare center; NEEP, Nutrition Embedded Evaluation Program; NEEP-IE, Nutrition Embedded Evaluation Program Impact Evaluation.

Small effects of the intervention were found on the likelihood of caregivers reporting the CBCC being open over the 5 d before the survey (DID: 0.31; SE: 0.15; *P* < 0.05) and on the number of meal days provided (DID: 0.51; SE: 0.12; *P* < 0.001), although the number of days when meals were provided was still low (<2 d/wk, on average). No effects were found on CBCC enrollment or attendance; a substantial decrease from high baseline levels occurred in both study arms during the study (**Table 3**). Analysis of CBCC-level data showed that, in the intervention group, 16 of 30 centers (53%) provided a meal the day before the survey, compared with 9 of 30 (30%) in the control group. CBCCs in the intervention group also provided more food and more nutritious meals than those in the control group (**Figure 3**).

**TABLE 3:** CBCC meal provision, enrollment, and attendance and adjusted DID impact estimates in children aged 36–72 mo at baseline living in treatment and control communities in Zomba district, Malawi: NEEP-IE study^1^

	Treatment (*n* = 660)		Control (*n* = 648)		DID	
Indicator	Baseline	Endline	Baseline	Endline	Impact	SE
Days center was open in last 7 d, *n*	3.91	3.09	4.08	2.95	0.29**	0.15
Center provided meals, %	23	46	29	40	10.6 pp**	3.5
Days meal provided in last 7 d, *n*	0.75	1.47	0.92	1.13	0.51***	0.12
Center enrollment, %	92	64	93	60	4.6 pp*	2.7
Center attendance in last 7 d, %	71	49	66	48	−4.0 pp	3.1

^1^All unadjusted baseline and endline values are means or percentages. Values for CBCC meal provision, enrollment, and attendance are unadjusted means at baseline and after 12 mo in the intervention and control groups. *P < 0.10, **P < 0.05, ***P < 0.001. DID, difference-in-difference; NEEP-IE, Nutrition Embedded Evaluation Program Impact Evaluation; pp, percentage points.

### Impact on child dietary intake and anthropometric measures

The intervention improved the dietary intake of foods consumed at home (**Table 4**), measured over a 24-h recall period, by preschoolers for energy, protein, and all micronutrients studied (zinc, iron, and vitamins A, C, B-6, and B-12). The intervention also improved mean dietary diversity in preschoolers, driven by the higher likelihood of intake of fruits and fish in the past 24 h (not reported in Table 4). No differences between girls and boys were found. For younger siblings, only endline data were collected on dietary diversity. Findings showed that mean DDS was 0.31 points greater in the intervention group (*P* < 0.05; mean DDS = 3.24 in the intervention group compared with 2.93 in the control group; data not reported). This difference was driven by the higher likelihood of the consumption of nuts, pulses, fruits, and vegetables. Moreover, the intervention group had a higher percentage of younger siblings who had achieved MDD in the past 24 h (39% in the intervention group compared with 28% in the control group; mean ± SE difference: 0.11 ± 0.05; *P* < 0.05).

**TABLE 4:** Unadjusted mean daily dietary intake at home measured by quantitative 24-h recall at baseline and after 12 mo in the intervention and control groups and adjusted DID impact estimates in children aged 36–72 mo at baseline living in treatment and control communities in Zomba district, Malawi: NEEP-IE study^1^

	Treatment (*n* = 606)		Control (*n* = 604)		DID	
Indicator	Baseline	Endline	Baseline	Endline	Impact	SE
Food quantity, g	566	846	595	720	153***	28.27
Energy, kcal	1273	1627	1321	1376	294***	50.30
Protein, g	40	54	42	48	8.12**	2.64
Iron, mg	11	13	11	12	1.64**	0.52
Zinc, mg	6	7	6	6	1.09**	0.33
Vitamin A, µg RAE	449	930	600	1013	59.44	71.73
Vitamin C, mg	47	101	65	99	19.72**	6.40
Vitamin B-6, mg	1.08	1.48	1.18	1.32	0.26***	0.06
Vitamin B-12, µg	0.53	0.92	0.66	0.73	0.31**	0.16
Individual dietary diversity score	5.35	5.80	5.42	5.51	0.36***	0.09
Individual food variety score	7.22	7.60	7.04	6.86	0.55***	0.15

^1^All unadjusted baseline and endline values are means. **P < 0.05, ***P < 0.001. DID, difference-in-difference; NEEP-IE, Nutrition Embedded Evaluation Program Impact Evaluation; RAE, retinol activity equivalents.

The intervention had no impact on linear growth in preschoolers; anthropometric indexes were relatively unchanged throughout the 12-mo period in treatment and control groups (**Table 5**). However, positive effects on HAZ (DID: 0.44; SE: 0.16; *P* < 0.05) with a concurrent reduction in the prevalence of stunting [DID: –17 percentage points (pp); SE: 6 pp; *P* < 0.05] were found in the younger siblings (*n* = 304). No effects were observed on WHZ during the 12-mo study period. This is not surprising because WHZ was close to the reference standards and wasting prevalence was very low in this sample (1–4%).

**TABLE 5:** Unadjusted mean HAZ, WAZ, and WHZ and the prevalence of stunting, underweight, and wasting at baseline and after 12 mo in the intervention and control groups and adjusted DID estimates for these indicators in children aged 36–72 mo and 6–24 mo at baseline living in treatment and control communities in Zomba district, Malawi: NEEP-IE study^1^

	Treatment (*n* = 155)		Control (*n* = 149)		DID	
Indicator	Baseline	Endline	Baseline	Endline	Impact	SE
Age 36–72 mo						
* n*	631		617			
HAZ	−1.75	−1.70	−1.74	−1.70	0.05	0.05
Stunted (HAZ <2 SDs), %	40	36	39	36	−1 pp	2.6
WAZ	−1.08	−1.16	−1.05	−1.15	0.05	0.05
Underweight (WAZ <2 SDs), %	17	34	17	32	2 pp	0.03
WHZ	0.09	−0.06	0.11	0.08	−0.04	0.07
Wasted (WHZ <2 SDs), %	1	1	1	2	−1 pp	0.01
Age 6–24 mo						
* n*	155		149			
HAZ	−1.70	−1.87	−1.61	−2.29	0.44**	0.16
Stunted (HAZ <2 SDs), %	41	45	42	63	−17 pp**	5.8
WAZ	−0.68	−1.05	−0.73	−1.18	−0.02	0.14
Underweight (WAZ <2 SDs), %	14	16	13	22	−5 pp	0.04
WHZ	0.12	0.04	0.09	0.09	−0.13	0.15
Wasted (WHZ <2 SDs), %	1	2	3	1	4pp	0.02

^1^All unadjusted baseline and endline values are means or percentages. **P < 0.05. DID, difference-in-difference; HAZ, height-for-age *z* score; NEEP-IE, Nutrition Embedded Evaluation Program Impact Evaluation; pp, percentage points; WAZ, weight-for-age *z* score; WHZ, weight-for-height *z* score.

## Discussion

The NEEP-IE study is, to our knowledge, the first CRCT to explicitly evaluate the impact of an integrated agriculture-nutrition intervention implemented through an ECD platform on household and children's nutrient intakes, dietary diversity, and anthropometric measures. Despite the short 12-mo time frame, the analysis found important benefits of the intervention that extended beyond the CBCC, improving several nutrition-related outcomes at the household level as well as among preschoolers and their younger siblings.

The analysis showed that the intervention increased caregiver knowledge of food groups and the role that food groups have in providing a balanced diet. Analysis of 24-h dietary recall data showed substantive improvements in preschool children's energy, protein, and micronutrient intake, including iron, zinc, and vitamins C, B-6, and B-12, as well as improved dietary diversity and higher frequency of intake of fruits and fish. Effect sizes varied, ranging from an equivalent of 13% of the RDA for iron to 52% of the RDA for protein intake. Because these estimates do not include contributions from the CBCC meals, they are likely to underestimate the overall treatment effect, even though the CBCC meals were not provided regularly. Benefits extended to younger siblings with positive effects of the intervention on dietary diversity, an effect driven by increased likelihood of consumption of nuts, pulses, fruits, and vegetables. Younger siblings in the intervention group were also more likely to have received a minimum of 4 food groups in the 24 h before the survey than children in the control group.

**FIGURE 4:**
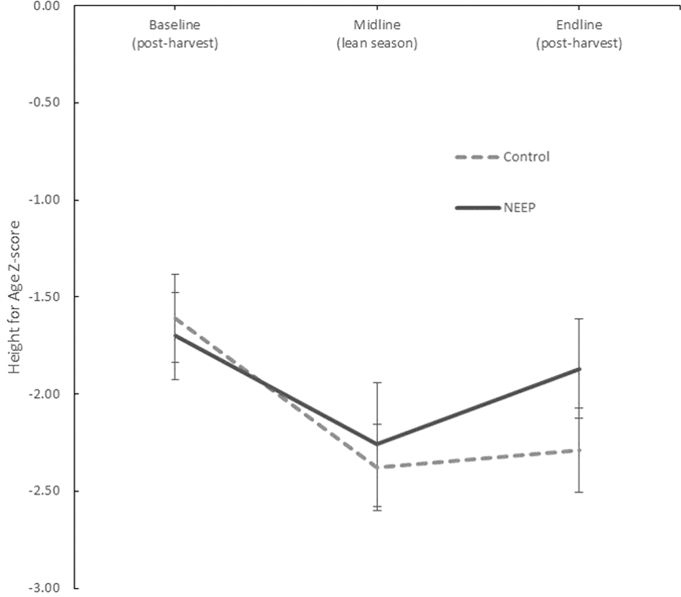
Baseline, midline, and endline unadjusted mean HAZ scores (with 95% CIs) by study group in children aged 6–24 mo at baseline, Zomba district, Malawi: the NEEP-IE study. Baseline *n* = 304, midline *n* = 244, endline *n* = 244. HAZ, height-for-age *z* score; NEEP, Nutrition Embedded Evaluation Program; NEEP-IE, Nutrition Embedded Evaluation Program Impact Evaluation.

No impacts were found on anthropometric indicators in preschoolers. However, a significant and large effect of the intervention was found in their younger siblings, including a smaller decline in HAZ between baseline and endline in the intervention group compared with the control group (difference equivalent to +0.44 SDs) and a smaller increase in stunting between baseline and endline in the intervention group than in the control group (equivalent to a difference of +17 pp). This finding is surprising given the short duration of the intervention (12 mo) and the fact that stunting is a cumulative process. As a robustness check for this result we examined the anthropometric data from the midpoint measurement after 6 mo of intervention. The midline data point coincides with the peak lean season when households in the study population face the highest levels of food insecurity. This analysis confirmed the protective effect of the intervention on linear growth in younger siblings. Although, in the control group, children's HAZ declined substantively during the first 6 mo and stabilized thereafter, the decline was initially less marked in the intervention group than in the control group at midline, with HAZ scores then improving considerably between midline and endline (**Figure 4**, **Supplemental Table 5**). The period between midline and endline coincides with the postharvest season when nutritious foods planted in intervention areas would have boosted household food availability.

The plausibility of these effects on linear growth in younger siblings is supported by improvements along hypothesized pathways of impact, including improvements in the following: household production of a range of nutritious foods (including an increased number of chickens and production of eggs) and diversity of production at the farm level, shifting the balance of food production toward a more nutritious bundle of crops; caregiver's nutrition knowledge; and child dietary diversity (including increases in the likelihood of consumption of nuts, pulses, fruits, and vegetables).

The intervention had small effects on the likelihood of CBCCs being open and on the frequency of the CBCCs’ provision of meals. CBCC meals in intervention areas did include more food and had a more nutritious balance than CBCC meals in the control group. The number of days when meals were provided, however, was still low, highlighting the need for further improvements. The intervention also had no impact on CBCC enrollment or attendance, which decreased in both groups throughout the study period. This unexpected result could be explained by the generally low frequency of meal provision found in the CBCCs, offering little or no extra incentive for preschool children's participation.

Nonetheless, the overall program impacts along the program impact pathways may have combined to provide a protective environment for households in the intervention group, with an emphasis on improving household access to nutritious foods and the nutrient density of meals at a critical age when young children are introduced to complementary foods. In the context of high levels of food insecurity witnessed during the study period (34), these factors may explain the relatively large magnitude of the effect on stunting. Two other studies on BCC aimed at improving dietary diversity and consumption of animal-source foods among infants and young children found similar effect sizes on stunting (35, 36). A CRCT in Peru evaluated the impact of health service–based nutrition education on feeding practices, dietary intake, and growth over 2 y. The intervention increased intake of animal-source foods at 6 and 8 mo, mean energy intake from complementary foods, and intake of micronutrients. At 18 mo, intervention-group children were 1 cm taller and 3 times less likely to be stunted compared with children in the control group (35). A CRCT in China comparing an educational intervention on complementary feeding to a control without an intervention found improved food diversity, meal frequency, and hygiene practices in the treatment group compared with the control group, as well as gains in length (0.66 cm) and weight (0.22 kg) at 1 y of age (36).

This study has several strengths, including the CRCT design and use of program impact pathways to assess plausibility of findings. There were also some important limitations. First, our sample for the impact on stunting on younger siblings included, by design, only caregivers who had both a preschool-age child and a younger child (aged <24 mo). This result is therefore not representative of the broader population of mothers with a child aged 6–24 mo but rather is representative of those who have both a preschooler and a younger child aged 6–24 mo. Second, the study population includes only 1 district in Malawi and, as such, has potentially limited external validity. However, the study villages were selected on the basis of food security conditions that are prevalent across much of the region and include a range of agro-ecological zones; thus, the evidence generated in this study is likely broadly relevant across the region. Moreover, the age distribution in this study is comparable to that found at the country level from the latest DHS (13). Another important limitation involves the issue of multiple hypothesis testing (37). There are several points to consider in terms of the rationale for reporting on multiple outcomes in this trial. First, the program was complex and involved a package of several interventions with various potential impact pathways across agriculture and nutrition domains. As per protocol, in this theory-driven evaluation, we evaluated the impact of the intervention on a set of primary outcomes that were expected to be affected by the program's different intervention components. In this article we report on impacts from the agriculture component, which was expected to improve household food production and production diversity; and from the nutrition BCC, training on meal planning, preparation, and safety, and the meals provided at the CBCC, which were expected to improve children's diets and nutrient intake. Thus, statistical tests were performed on outcomes along the main program impact pathways to assess the mechanisms through which the complex intervention worked (38). In this study, there is evidence of significant effects across all the nutrition-related program impact pathways, which is reassuring and consistent with the rationale and design of the intervention activities. To explore this issue with regard to impact on nutrient intake, we examined the impact of the intervention on the mean probability of adequacy of nutrient intake in preschoolers, an aggregate metric for quality of diet, and found results consistent with those presented here. Another limitation relates to the measurement error, including respondent and enumerator bias, in the 24-h dietary assessment measured by recall (24). To mitigate this, we included questions related to intake in different sections of the questionnaire to allow triangulation between different individual- and household-level data. In addition, to estimate usual intake, the 24-h dietary assessment was repeated on nonconsecutive days for a subset of households at endline (20%). Because of budget constraints we were not able to include any measurements of biomarkers for micronutrient status or infection.

In conclusion, this study suggests that community-owned ECD centers can be an effective platform to deliver agriculture and nutrition interventions and achieve improvements in household production diversity, maternal knowledge of child nutrition, and preschool children's diets while also benefiting their younger siblings’ dietary diversity and linear growth. The study findings highlight that community-based ECD centers can provide a platform to change household behaviors related to food production and consumption, influencing decisions that may benefit all household members at different lifecycle stages. Moreover, evidence from this study indicates that the intervention had a protective effect during a period of high food insecurity, suggesting a role for these types of interventions within social protection portfolios. The intervention relies on community contributions and may provide a sustainable option for government scale-up.

## Supplementary Material

Supplemental Files

## Notes

Supported by the CGIAR Research Program on Agriculture for Nutrition and Health (A4NH), led by the International Food Policy Research Institute (IFPRI). Also supported by the Nutrition Embedding Evaluation Program (NEEP) grant from PATH and the Innovative Methods and Metrics for Agriculture and Nutrition (IMMANA) grant, both funded by the UK government's Department for International Development.

This study was implemented in a partnership between IFPRI, Save the Children, and Chancellor College, University of Malawi.

Author disclosures: AG, AM, MS, NR, AT, MK, HM, HA, and MR, no conflicts of interest.

Supplemental Tables 1–5 are available from the “Supplementary data” link in the online posting of the article and from the same link in the online table of contents at https://academic.oup.com/jn/.

**Abbreviations used**
AEDO: agriculture extension development officer
BCC: behavior change communication
CBCC: community-based childcare center
CRCT: cluster-randomized controlled trial
DDS: dietary diversity score
DHS: Demographic and Health Survey
DID: difference-in-difference
ECD: early childhood development
HAZ: Height-for-age *z* score
IYCF: infant and young child feeding
MDD: minimum dietary diversity
NEEP-IE: Nutrition Embedded Evaluation Program Impact Evaluation
pp: percentage point
SC: Save the Children
WAZ: weight-for-age *z* score
WHZ: weight-for-height *z* score
