# Supplementary material for: Using a Community-Based Early Childhood Development Center as a Platform to Promote Production and Consumption Diversity Increases Children's Dietary Intake and Reduces Stunting in Malawi: A Cluster-Randomized Trial
Source: J Nutr. 2018 Sep 10;148(10):1587–97. doi: 10.1093/jn/nxy148 (PMC6168702; doi:10.1093/jn/nxy148)
Supplement: Supplemental Files [file nxy148_supplemental_files.zip › Gelli_NEEPIE_JoN_12062018_NTC.docx]

**Using a community-based early childhood development center as a platform to promote production and consumption diversity increases children’s dietary intake and reduces stunting in Malawi: A cluster randomized trial.**

Aulo Gelli^1*^, Amy Margolies^2^, Marco Santacroce^1^, Natalie Roschnik^3^, Aisha Twalibu^3^, Mangani Katundu ^4^, Helen Moestue^3^, Harold Alderman^1^, and Marie Ruel^1^.

^*^ Corresponding author
Email: [a.gelli@cgiar.org](mailto:a.gelli@cgiar.org)

^1^ International Food Policy Research Institute (IFPRI), 2033 K Street NW, Washington, DC 20006, USA.

^2^ Johns Hopkins University, USA.

^3^ Save the Children, USA/Malawi.

^4^ Chancellor College, University of Malawi, Malawi.

Other author emails:

Amy Margolies ([margolies.amy@gmail.com](mailto:margolies.amy@gmail.com))

Marco Santacroce ([m.Santacroce@cgiar.org](mailto:m.Santacroce@cgiar.org))

Natalie Roschnik ([n.roschnik@savethechildren.org.uk](mailto:n.roschnik@savethechildren.org.uk))

Aisha Twalibu ([aisha.twalibu@savethechildren.org](mailto:aisha.twalibu@savethechildren.org))

Mangani Katundu ([manganikatundu@gmail.com](mailto:manganikatundu@gmail.com))

Helen Moestue ([hmoestue@savechildren.org](mailto:hmoestue@savechildren.org))

Harold Alderman ([h.alderman@cgiar.org](mailto:h.alderman@cgiar.org))

Marie Ruel ([m.ruel@cgiar.org](mailto:m.ruel@cgiar.org))

Authors’ last names for PubMed indexing: Gelli, Margolies, Santacroce, Roschnik, Twalibu, Katundu, Moestue, Alderman, Ruel.

Word count: 5584

Number of figures: 4

Number of tables: 5

Running title: Impact of agriculture-nutrition intervention in Malawi.

*OSM submitted: Supplemental Tables 1, 2, 3, 4, 5, qualitative research findings.

+Sources of financial support: We acknowledge support from the CGIAR Research Program on Agriculture for Nutrition and Health (A4NH), led by IFPRI. This work was supported by the Nutrition Embedding Evaluation Program (NEEP) grant from PATH, and the Innovative Methods and Metrics for Agriculture and Nutrition (IMMANA) grant, both funded by the UK government’s Department for International Development. The views expressed do not necessarily reflect the UK government’s official policies.

Conflict of interest: The authors have no conflict of interest.

# Abbreviations: AEDOs-Agriculture Extension Development Officers; ABCC– Behavior change communication; CBCC–Community based childcare center; CRCT–Cluster randomized control trial; DDS–Dietary Diversity Score; DID–Difference-in-difference; ECD–Early childhood development; FVS–Food variety score; HAZ–Height-for-age z-score; HH–Household; IFPRI–International Food Policy Research Institute; IYCF–Infant and young child feeding; kg–kilogram; MWK–Malawian Kwacha; MDD–Minimum dietary diversity; NEEP-IE–Nutrition Embedded Evaluation Program Impact Evaluation; pp–percentage point; OFSP–Orange-fleshed sweet potato; RDA–Recommended Dietary Allowance; SC- Save the Children; SD–Standard deviation; WAZ–Weight-for-age z-score; WHZ–Weight-for-height z-score.

**Abstract**

**Background**: Children in Malawi face nutritional risks related to low-quality diets and chronic malnutrition.

**Objective**: This study evaluated the impact of a one-year early childhood development (ECD) center-based agriculture and nutrition intervention aimed at improving household production diversity, maternal knowledge on child nutrition and feeding practices, and children’s diets and anthropometry.

**Methods**: A longitudinal cluster randomized control trial was implemented in 60 community-based childcare centers (CBCCs), covering 1,248 preschool children (aged 36–72mo) and 304 younger siblings (6–24mo). CBCCs were randomized to 1) control group providing Save the Children’s ECD program; or 2) treatment group providing standard ECD program with additional activities to improve nutritious food production and behavior change communication (BCC) to improve diets and care practices for young children. Primary outcomes were household production and production diversity, preschooler enrollment and attendance, and dietary intake measured by quantitative 24-h recall and minimum diet diversity for younger siblings. Secondary outcomes included anthropometry for preschoolers and younger siblings, child development scores for preschoolers, and women’s asset ownership and time use (latter two not discussed in this paper). We used difference-in-difference (DID) estimates to assess impacts.

**Results**: Compared to the control group, preschool children in the intervention group had greater increases in nutrient intakes and in dietary diversity. No impacts on anthropometry were seen in preschoolers. Younger siblings in the intervention group had greater increases in height-for-age z-scores than children from the control group (DID 0.44, P<0.05) and greater reductions in the prevalence of stunting (DID -17pp, P<0.05). The plausibility of the impact on growth in younger siblings was supported by effects along program impact pathways, including production of nutritious foods, caregiver knowledge, and dietary diversity.

**Conclusions**: Implementing an integrated agriculture and nutrition intervention through an ECD platform benefitted children’s diets and reduced stunting among younger siblings of targeted preschoolers.

Trial registered on the ISRCTN Registry as ISCRCTN96497560.

**Keywords**: Early childhood, nutrition, diets, agriculture, impact evaluation.

# Introduction

Estimates of the global burden of malnutrition indicate that undernutrition causes over 3 million child deaths per year [1] and that 155 million children under five years of age are stunted [2]. Deficiencies in micronutrients also contribute to increased child and maternal mortality, while impairing children’s physical and mental development [3].

Reviews of the contributions of nutrition-sensitive development, and agricultural programs in particular, conclude that although such programs have the potential to improve nutrition, this potential is yet to be fully realized [4; 5]. Limitations in the design and implementation of nutrition-sensitive agricultural interventions, as well as the lack of rigor in impact evaluations, prevent clear conclusions regarding their contribution in improving nutrition [5]. More recent evidence suggests that well-designed and carefully implemented nutrition-sensitive agricultural programs improve maternal and child nutrition and are effective at increasing intake of nutritious foods and improving diet quality when they include a strong behavior change communication (BCC) and women’s empowerment interventions [6]. Early childhood development (ECD) programs are another platform recommended for delivering nutrition interventions to preschool children [5]. One justification for using ECD platforms to deliver nutrition interventions is the potential for synergies between ECD and nutrition on both child development and nutrition outcomes [7; 8]. Another justification is that, with the recognition of the importance of the period from conception to the child’s second birthday, the focus of nutrition programs has shifted to this period, and as a result, children two to six years old (preschoolers) are left out of many nutrition and health programs until they enroll in school. While preschool children may have less potential to benefit from nutrition interventions in terms of linear growth, they still have nutritional needs, including receiving a nutritious and healthy diet that allows them to meet their nutrient requirements. Integrated ECD and nutrition investments can thus provide a way to maintain a continuum in nutrition programming among children beyond the first two years. Moreover, ECD programs can be leveraged to reach caregivers and promote healthy diets among all other household members, including younger siblings.

The national ECD program in Malawi is led by the Ministry of Gender, Children and Social Welfare and consists of support to preschools (known as Community-Based Childcare Centers (CBCCs)) and parenting groups. CBCCs are community-led centers that promote child development by providing safe and stimulating environments, access to health and nutrition services, and training for parents and caregivers. CBCCs service 3–6-year-olds and are open from 8 a.m. to 11 a.m., five days a week. When possible, a porridge is provided midmorning with food contributions from the community. However, irregular supply of food has been reported as one of the main causes of child absenteeism and CBCC closure [9].

The Nutrition Embedded Evaluation Program Impact Evaluation (NEEP-IE) used a cluster randomized control trial (CRCT) design to examine the effectiveness of using a community-based ECD center as a platform to: promote household production and consumption diversity; improve caregiver knowledge and practices of nutrition and infant and young child feeding (IYCF) practices; and improve diets and nutrition among preschoolers and their younger siblings [10]. This study presents the impacts on all the primary outcomes of the trial and the nutrition-related secondary outcomes (anthropomterics). Impacts on the other secondary outcomes, including preschoolers’ cognitive development and women’s asset ownership, time use and productivity were also assessed and will be reported in separate publications.

By testing the effectiveness of the intervention through community-based ECD centers, this study addresses an evidence-base gap on innovative delivery platforms for nutrition sensitive interventions [11]. Though the primary target group in this study was preschoolers, the intervention involved their parents and caregivers as entry points to influence household decisions and potentially reach younger siblings during a critical period for their growth. Through the analysis of intermediate outcomes along theorized program impact pathways, the study aimed to establish plausibility of findings and identify the channels through which impacts may have been achieved [12].

# Methods

## Country context

Malawi has one of the highest rates of chronic malnutrition in the world, with 37% of children aged 6–59mo moderately or severely stunted [13]. Severe climatic shocks and flooding in 2014–16 resulted in high levels of food insecurity across the country, leaving 2.8 million people in need of humanitarian support [14; 15]. The situation worsened the following year with widespread drought [16]. After several years of agriculture-led growth and consistent improvements in health and nutrition indicators [17], these shocks reversed momentum and risked serious long-term negative effects on the population’s health and nutrition.

## Intervention description

### The standard package

Save the Children (SC) has supported CBCCs in Zomba district of Malawi since 2008. The standard ECD package provided to SC-supported communities is based on materials developed by the Government of Malawi. As part of this study, caregivers in all the CBCCs received a two-week training provided by government-approved trainers who conducted counseling sessions using a government manual. The topics discussed included child nutrition and stimulation, and parental role in school readiness. Caregiver groups were led by the trained facilitators once a month for the study duration. As part of the training, links between CBCCs and parenting groups were strengthened with the aim of improving parenting practices and also reaching younger siblings.

### The integrated intervention

The NEEP-IE integrated agriculture and nutrition intervention aimed at increasing the effectiveness of the government ECD program. The agriculture component promoted improved production of nutritious foods and food diversification by using CBCC gardens as a demonstration site for communities. Before the two main planting seasons, government agriculture extension development officers (AEDOs) held three days of training for parents, CBCC Management Committee representatives, farmers, and community agents on land preparation, selection of nutritious crops, agriculture production techniques, pest and disease management, manure-making and application, harvesting, storage, and processing, and chicken rearing. Village savings and loans groups were also supported by SC to start home gardens and help communities purchase supplies for CBCC meals. The agriculture training focused on nutritious food production, including a traditional variety of orange maize (rich in vitamin A) and biofortified orange-fleshed sweet potato (OFSP), legumes and nuts (soya beans, pigeon peas, cowpeas, and groundnuts), and green leafy vegetables (amaranthus), as well as care for chickens. Participating households received seeds along with 10 chicks.

The nutrition component was aimed at improving feeding and caring practices, engaging parents and other caregivers in the planning and preparation of meals in CBCCs. Activities included BCC and training on nutritional needs of infants and young children, year-round meal planning and preparation, food storage, hygiene, waste disposal, and monitoring of meal provision. Recipes included preparation of nutrient-rich meals based on seasonal foods. CBCC Management Committee members, CBCC caregivers (teachers), lead farmers, and parents received a three-day nutrition training by Government AEDOs and nutrition assistants. By taking turns preparing CBCC meals throughout the study period, parents continued to practice new recipes at the CBCC, which they then replicated at home. The first set of agriculture training sessions was implemented after the baseline survey in December 2015, before the planting season. The nutrition trainings began in February 2016. Monthly follow-up visits were undertaken by AEDOs and Save the Children staff.

## Program theory

The program theory for the integrated agriculture and nutrition intervention was guided by the Lancet Series framework on Maternal and Child Nutrition [1] and the framework describing pathways by which agriculture can improve nutrition [18] through three channels (**Figure 1**). First, the intervention could affect agriculture by increasing production, improving the household-level availability of nutritious foods. Second, the nutrition BCC could improve diets and feeding practices by improving caregiver knowledge. And third, by increasing the regularity and quality of CBCC meals, the intervention could influence CBCC participation, possibly enhancing both their learning and nutritional status [19].

## Study design and participants

A CRCT was implemented in 60 rural communities with CBCCs supported by SC program in Zomba district, Malawi. The CRCT study protocol is published elsewhere [10]. The evaluation combined quantitative and qualitative methods with two rounds of surveys timed 12mo apart. Communities were randomly assigned to one of two arms (**Figure 2**):

1. Control group: Communities with CBCCs supported by SC ECD program
2. Intervention group: Communities with CBCCs supported by SC ECD program with additional agriculture and nutrition intervention

The intervention was implemented in 30 of the 60 rural communities after the baseline survey. Several reasons explain why the control group in this case was not a control without intervention. The Government of Malawi is committed to scaling up the ECD support activities across all CBCCs and an impact evaluation on the cost-effectiveness of different ECD strategies is underway. This study complemented ongoing work by examining the relative impact of alternative models, focusing on how to enhance participation in the CBCCs and support nutrition of children at a critical age in their development.

The 60 CBCCs were randomly selected in two stages from a pool of 235 CBCCs located in 47 primary school clusters assisted by SC. Due to the clustering of the CBCCs around primary schools, the list of 235 CBCCs was screened to flag clusters where more than one CBCC was supported. The cluster (unit of randomization) was the primary school cluster that included several CBCCs. Twenty-seven clusters with ongoing training activities in more than one CBCC were excluded from the first stage of randomization to minimize possible contamination. Twenty clusters were then randomly assigned to 2 groups of 10 clusters, where randomization was stratified geographically across three traditional authority areas. In the second stage of randomization, 3 CBCCs were selected at random within each cluster. As 6 clusters had fewer than 3 CBCCs, a larger number of CBCCs was randomly selected from 3 other clusters to allow selection of a full sample of 30 CBCCs per arm. The number of CBCCs per cluster ranged from 1 to 6 (mean=3.5 and median=3). The random allocation was undertaken using the “sample” command in STATA with random seed set to the serial number of the first currency bill drawn from the first author’s wallet. Enumerators were not blinded to the allocation.

The study targeted all children aged 6–72mo and their caregivers in the 60 communities. The primary reference group included children aged 36–72mo at baseline (preschooler group) living in the service area of SC‐supported CBCCs. A secondary reference group included all children 6–24mo at baseline (younger sibling group) living in households with at least one other child in the preschooler group. Primary outcomes included household food production and production diversity; individual dietary intake and dietary diversity score (DDS) (in preschoolers); CBCC enrolment and attendance (in preschoolers); and DDS and minimum dietary diversity (MDD) (in younger siblings). Secondary outcomes included anthropometry (weight-for-age, height-for-age, and weight-for-height z-scores (WAZ, HAZ, and WHZ, respectively) for all children 6–72mo; child development in preschoolers (not reported in this paper) and women’s asset ownership, time use and productivity (not reported in this paper). The scope of this study is limited to the analysis and reporting of all the primary study outcomes and the single secondary outcome (anthropometrics) per protocol that are most relevant to nutrition. The two remaining secondary outcomes, including child development and women’s asset ownership, time use and productivity, will be the focus of a set of similar analyses that will be published separately in thematic journals.

## Sample sizes

Based on initial power calculations and resource availability, we originally planned for 30 clusters (CBCCs) per treatment arm, with 20 households in each cluster to identify reasonable treatment impacts of the intervention on the primary outcomes. However, after preliminary community visits prior to the baseline survey, the original sampling strategy was modified to account for the implementation approach adopted by SC involving the clustering of CBCCs around primary schools. Adjusting for intra-cluster correlation coefficients at the primary school cluster level, where 60 CBCCs were clustered into 2 groups of 10 primary school clusters with 3 CBCCs each, would provide 80% power to detect a 0.4 standard deviation (SD) difference in the individual DDS between treatment groups at the 5% significance level. The sampling of households was conducted through a census within a catchment area for each CBCC. Households with children in the preschool reference age group were then randomly selected for study participation.

## Data collection

The baseline and endline surveys were completed in December 2015 and December 2016, respectively. Anthropometry was also collected at midline in April 2016.

### Household food production

Agricultural production was estimated for each crop cultivated in the previous 12 months during the household interview. A production diversity index was calculated as a count of the number of food groups produced during the previous 12 months (12 food groups were included; the scale scores ranged from 0 to 12) identical to that used to measure dietary diversity [20, 21]. A production variety index was computed as the total count of the number of individual crops that households reported cultivating during that same period.

### Caregiver IYCF knowledge and practices

Knowledge of IYCF was assessed through caregiver recall in two ways. We first elicited knowledge by asking caregivers open-ended questions on what they knew about the nutritional needs of infants (0–6mo) and young children (separately for 6–24mo and 2–5y age groups) (e.g., “What do you know about the needs of children 0–6mo regarding feeding?”). We subsequently asked caregivers a battery of questions on knowledge of specific practices (e.g., “How long after birth should a baby start breastfeeding?”) as described in WHO guidelines [22]. Caregivers were also asked about knowledge of food groups and of the properties of foods within different food groups. Open-ended question responses were coded and analyzed both individually and by generating a food group knowledge score that aggregated responses of relevant questions (with scores ranging from a minimum of 0 to a maximum of 6).

### Children’s diets, IYCF, and anthropometry assessment

Dietary assessment was undertaken using the interactive multi-pass 24-hr recall method [23]. To estimate the distribution of usual intake and account for within-person variation [24], a subset of 120 households was selected to have two 24-hr recalls at least two days apart. Prior to the recall interview, caregivers were briefed on the purpose and methods of interview. Interviews were conducted using visual aids to assist in estimating portion sizes. Individual recipes were broken down into individual ingredients at household level. A preferred method was established for each food, including direct weighing, standard portion sizes, and calibrated portion size models. Quantities in grams of different food items that children had consumed over the last 24 hours were converted into nutrients using a food composition table adapted for Malawi [25], adjusting for nutrient retention factors of cooked foods [26]. Outliers in caloric intake with values over 3 SDs were excluded from the nutrient intake analysis. The assessment did not include meals provided in CBCCs, as caregivers at home were in most cases not aware of specific details of meals provided in CBCCs, or actual quantities consumed by children. Meal provision data were collected at CBCCs, including recipes, ingredients, and quantities provided per child, though these were not included in the child-level estimates of food intake. Child dietary diversity (for preschoolers) and household dietary diversity were measured using the DDS, calculated as a count of the number of food groups consumed by children in the 24-hr assessment [20; 21] and in a household food consumption 7-day recall [27]. Twelve food groups were included (the scale of the scores was 0 to 12). It is important to note that no dietary diversity indicator has been validated for preschool children; current validated dietary diversity indicators exist only for children 6–24mo [22] and for adult women [28]. At the household level, we also used the household food variety score (FVS), calculated as a count of the number of individual food items that households reported consuming in the previous week [21]. At endline, we included the WHO MDD indicator for younger siblings [22]. The MDD score was calculated as the prevalence of children consuming a count of four or more of seven food groups during the previous 24 hours as per WHO guidelines. Anthropometric data included measurements of height and weight for all children from 6mo to 72mo undertaken during home visits. Recumbent length of children <2y and standing height of children >2y was measured to the nearest 0.1 cm using portable fixed base stadiometers or length boards; weight was measured to the nearest 0.1 kg using electronic scales. All enumerators collecting anthropometric data were trained using standard WHO guidelines and measurements [29] were practiced before the survey through standardization exercises. From these standardization sessions inter- and intra-observer variation of measurement error was documented and the necessary corrections to procedures were made. All measurements were undertaken by an anthropometrist and an assistant. Linear growth was examined using the HAZ and prevalence of stunting. HAZ, WAZ, and WHZ scores were calculated using the 2006 WHO growth standard using WHO cut-offs [29]. Stunting was defined as HAZ < -2 SD, wasting as WHZ < -2 SD, and underweight as WAZ <-2 SD. Recommended Dietary Allowance (RDA) were obtained from [30, 31]. All data (including dietary assessments) were collected by trained enumerators using electronic, android-based tablets with computer-assisted personal interview (CAPI) software. An additional back-check survey for quality assurance purposes was conducted in a random sample of 120 households. Ethical clearance was obtained from ethics boards at Chancellor College, U. of Malawi (Ref: NCST/RTT/2/6), and the International Food Policy Research Institute (IFPRI). Informed consent was obtained from parents through written and verbal information provided before interviews.

## Statistical analysis

The analysis followed an intention to treat approach as per the published protocol [10]. The impact on dietary intake in preschool children and anthropometry in children 6–72mo was assessed with a difference-in-difference (DID) estimator using multilevel regression models accounting for the hierarchical nature of the data [32]. The multilevel models used fixed effects and random effects at cluster and household level. The DID estimate was calculated as the average change in the outcome of interest between baseline and endline in the intervention arm minus the change in outcome in the control arm. The impact on the dietary diversity IYCF indicator in younger siblings was estimated using single differences at endline because no baseline information was available. Regression models for child-level indicators were adjusted for sex and age. The regressions used linear probability models for both continuous and binary variables for ease of interpretation, unless otherwise specified. Impacts were considered statistically significant at P<0.05. Robustness analysis for the anthropometry impact estimates in younger siblings included data from the midline survey. The robustness analysis also included comparing regression results for younger siblings at each time (“full sample”, n=304) to those measured in all three time points only (“full cohort”, n=208) The study was registered on the ISCRCTN registry (ISCRCTN96497560). As the allocation of clusters to study arms was random, following [33], significance tests of differences at baseline were not undertaken.

# Results

## Trial attrition

A total of 1,199 households and 60 CBCCs in Zomba district were surveyed at baseline. The endline survey included 1,122 households in 60 CBCCs, leading to a 7% attrition rate at household level. The main reason for attrition included households that moved out of the study area (64 households), other reasons included deaths (4 children) and refusals for re-interviews. The attrition rate was not significantly different across treatment groups nor was the probability of attrition correlated with treatment assignment. No statistically significant differences in means of dietary intake outcomes or HAZ between attrited and non-attrited children were found at baseline (**Supplemental Table 1**).

## Baseline characteristics and tests of balance

At baseline, average household size had 5.3 members and close to one in three households were female-headed. Thirty-five percent of household heads had completed primary education. Among mothers, only about 20% had completed primary education. The prevalence of child stunting was high (~40%), whereas wasting was almost nonexistent (1–3%), similar to the country-level status reported in the latest Demographic and Health Survey (DHS) [13]. On average, 26% of households had children in both preschool and younger sibling age groups, a similar rate to that in the latest DHS (27%). Examination of the age distribution in younger siblings at baseline indicated that approximately half were aged between 6–12mo and half between 12–24mo. CBCC participation was high, with over 90% of preschool children enrolled in a CBCC, and attendance rates were nearly 80% in the five days prior to the survey. However, only 26% of children reported receiving meals in CBCCs, and meals were provided on average for only one day out of five, highlighting a role for the intervention in increasing the regularity of meal provision. Overall, no substantive differences between intervention and control group were found in the baseline characteristics of the study population (**Table 1**).

## Impact on household food production and consumption

At household level, positive effects were observed on production diversity and on production of nutritious foods (biofortified OFSP, groundnuts, pigeon peas, and soya), as well on chickens owned and eggs produced in the three months prior to the survey (**Table 2**). Overall, production of these commodities was low at baseline, but significant increases were seen for products targeted by the intervention. No effects were found on household expenditures (including consumption from own production), suggesting that the intervention did not act as an income transfer. Positive effects were found on household dietary diversity (not shown), suggesting that the intervention resulted in households consuming different foods.

## Impact on caregiver knowledge

Positive effects were found when eliciting responses from open-ended questions on broad nutrition topics related to IYCF practices (**Supplemental Table 2**). However, when participants were asked questions on specific knowledge of IYCF practices, no effects of the intervention were found.

Positive impacts were also found on caregiver knowledge related to the importance of different food groups (**Supplemental Table 3**). These effects were driven by knowledge of foods considered important for growth (including beans and groundnuts) and foods needed for energy (including fats and oils). Further analysis of the sources of caregiver knowledge highlighted that SC (the implementer of the intervention) was the main knowledge source for messages on feeding practices (Supplemental Table 4).

## Impact on CBCC meals and participation

Small effects of the intervention were found on the likelihood of caregivers reporting the CBCC being open over the five days prior to the survey (DID 0.31, standard error (SE) 0.15, P<0.05) and on number of meal days provided (DID 0.51, SE 0.12, P<0.001), though the number of days when meals were provided was still low (less than 2d per week on average). No effects were found on CBCC enrolment or attendance; a substantial decrease from high baseline levels occurred in both study arms during the study (**Table 3**). Analysis of CBCC-level data showed that in the intervention group 16/30 centers (53%) provided a meal the day before the survey, compared to 9/30 (30%) in the control group. CBCCs in the intervention group also provided more food and more nutritious meals than those in the control group (**Figure 3**).

## Impact on child dietary intake and anthropometry

The intervention improved dietary intake of foods consumed at home (**Table 4**), measured over a 24-hr recall period, by preschoolers for energy, protein, and all micronutrients studied (zinc, iron, and vitamins A, C, B6, and B12). The intervention also improved mean dietary diversity in preschoolers, driven by the higher likelihood of intake of fruits and fish in the past 24 hours (not reported in table). No differences between girls and boys were found. For younger siblings, only endline data were collected on dietary diversity. Findings showed that mean DDS was 0.31 points greater in the intervention group (P<0.05, mean DDS=3.24 in the intervention group versus 2.93 in the control group, not reported); this difference was driven by the higher likelihood of consumption of nuts, pulses, fruits, and vegetables. Moreover, the intervention group had a higher percentage of younger siblings who had achieved MDD in the past 24 hours (39% in the intervention group versus 28% in the control group, mean difference=0.11, SE 0.05, P<0.05).

The intervention had no impact on linear growth in preschoolers; anthropometric indices were relatively unchanged throughout the 12-mo period in treatment and control groups (**Table 5**). However, positive effects on HAZ (DID 0.44, SE 0.16, P<0.05) with a concurrent reduction in the prevalence of stunting (DID -17pp, SE 6, P<0.05) were found in the younger siblings (n= 304). No effects were observed on WHZ during the 12-mo study period. This is not surprising since WHZ was close to the reference standards and wasting prevalence was very low in this sample (1–4%).

# Discussion

The NEEP-IE study is, to our knowledge, the first CRCT to explicitly evaluate the impact of an integrated agriculture–nutrition intervention implemented through an ECD platform on household and children’s nutrient intakes, dietary diversity, and anthropometry. Despite the short, 12-mo timeframe, the analysis found important benefits of the intervention that extended beyond the CBCC, improving several nutrition-related outcomes at the household level as well as among preschoolers and their younger siblings.

The analysis showed that the intervention increased caregiver knowledge of food groups and the role that food groups have in providing a balanced diet. Analysis of 24-hr dietary recall data showed substantive improvements in preschool children’s energy, protein, and micronutrient intake, including iron, zinc, and vitamins C, B6, and B12, as well as improved dietary diversity and higher frequency of intake of fruits and fish. Effect sizes varied, ranging an equivalent of 13% RDA for iron to 52% RDA for protein intake. As these estimates do not include contributions from the CBCC meals, they are likely to underestimate the overall treatment effect even though the CBCC meal were not provided regularly. Benefits extended to younger siblings, with positive effects of the intervention on dietary diversity, an effect driven by increased likelihood of consumption of nuts, pulses, fruits, and vegetables. Younger siblings in the intervention group were also more likely to have received a minimum of four food groups in the 24 hours prior to the survey than children in the control group.

No impacts were found on anthropometric indicators in preschoolers. However, a statistically significant and large effect of the intervention was found in their younger siblings, including a smaller decline in HAZ between baseline and endline in the intervention compared to the control group (difference equivalent to +0.44 SDs), and a smaller increase in stunting between baseline and endline in the intervention compared to the control group (equivalent to a difference of +17pp). This finding is surprising given the short duration of the intervention (12mo) and the fact that stunting is a cumulative process. As a robustness check for this result we examined the anthropometric data from the midpoint measurement after 6mo of intervention. The midline data point coincides with the peak lean season when households in the study population face the highest levels of food insecurity. This analysis confirmed the protective effect of the intervention on linear growth in younger siblings. While in the control group children’s HAZ declined substantively during the first six months and stabilized thereafter, the decline was initially less marked in the intervention group than in the control group at midline, with HAZ scores then improving considerably between midline and endline (**Figure 4, Supplemental Table 5**). The period between midline and endline coincides with the postharvest season when nutritious foods planted in intervention areas would have boosted household food availability.

The plausibility of these effects on linear growth in younger siblings is supported by improvements along hypothesized pathways of impact, including improvements in: household production of a range of nutritious foods (including an increased number of chickens and production of eggs) and diversity of production at farm level, shifting the balance of food production toward a more nutritious bundle of crops; caregiver’s nutrition knowledge; and child dietary diversity (including increases in the likelihood of consumption of nuts, pulses, fruits, and vegetables).

The intervention had small effects on the likelihood of CBCCs being open and on frequency of CBCCs’ provision of meals. CBCC meals in intervention areas did include more food and had a more nutritious balance than CBCC meals in the control group. The number of days when meals were provided, however, was still low, highlighting the need for further improvements. The intervention also had no impact on CBCC enrolment or attendance, which decreased in both groups throughout the study period. This unexpected result could be explained by the generally low frequency of meal provision found in the CBCCs, offering little or no extra incentive for preschool children’s participation.

Nonetheless, the overall program impacts along the program impact pathways may have combined to provide a protective environment for households in the intervention group, with an emphasis on improving household access to nutritious foods and the nutrient density of meals at a critical age when young children are introduced to complementary foods. In the context of high levels of food insecurity witnessed during the study period [34], these factors may explain the relatively large magnitude of the effect on stunting. Two other studies on BCC aimed at improving dietary diversity and consumption of animal-source foods among infants and young children found similar effect sizes on stunting [35; 36]. A CRCT in Peru evaluated the impact of health service-based nutrition education on feeding practices, dietary intake, and growth over two years. The intervention increased intake of animal-sourced foods at six and eight months, mean energy intake from complementary foods, and intake of micronutrients. At 18mo, intervention group children were 1cm taller and three times less likely to be stunted compared to children in the control group [35]. A CRCT in China comparing an educational intervention on complementary feeding to a control without intervention found improved food diversity, meal frequency, and hygiene practices in the treatment group compared to control, as well as gains in length (0.66cm) and weight (0.22kg) at one year of age [36].

This study has several strengths, including the CRCT design and use of program impact pathways to assess plausibility of findings. Some important limitations also arose: First, our sample for the impact on stunting on younger siblings included, by design, only caregivers who had both a preschool-age child and a younger child (<24mo). This result is therefore not representative of the broader population of mothers with a child 6–24mo, but rather is representative of those who have both a preschooler and a younger child 6–24mo. Second, the study population includes only one district in Malawi and as such has potentially limited external validity. However, the study villages were selected based on food security conditions that are prevalent across much of the region and include a range of agroecological zones; thus the evidence generated in this study is likely broadly relevant across the region. Moreover, the age distribution in this study is comparable to that found at country level from the latest DHS [13]. Another important limitation involves the issue of multiple hypothesis testing [37]. There are several points to consider in terms of the rationale for reporting on multiple outcomes in this trial. First, the program was complex and involved a package of several interventions with various potential impact pathways across agriculture and nutrition domains. As per protocol, in this theory-driven evaluation, we evaluated the impact of the intervention on a set of primary outcomes that were expected to be affected by the program’s different intervention components; in this paper we report on impacts from the agriculture component, which was expected to improve household food production and production diversity; and from the nutrition BCC, training on meal planning, preparation, and safety, and the meals provided at the CBCC that were expected to improve children’s diets and nutrient intake. Thus, statistical tests were performed on outcomes along the main program impact pathways to assess the mechanisms through which the complex intervention worked [38]. In this study, there is evidence of statistically significant effects across all the nutrition-related program impact pathways, which is reassuring and consistent with the rationale and design of the intervention activities. To explore this issue with regards to impact on nutrient intake, we examined the impact of the intervention on the mean probability of adequacy of nutrient intake in preschoolers, an aggregate metric for quality of diet, and found results consistent with those presented here. Another limitation relates to the measurement error, including respondent and enumerator bias, in the 24-hr dietary assessment measured by recall [24]. To mitigate this, we included questions related to intake in different sections of the questionnaire to allow triangulation between different individual and household-level data. In addition, to estimate usual intake, the 24-hr dietary assessment was repeated on nonconsecutive days for a subset of households at endline (20%). Because of budget constraints we were not able to include any measurements of biomarkers for micronutrient status or infection.

### Conclusions

This study suggests that community-owned ECD centers can be an effective platform to deliver agriculture and nutrition interventions and achieve improvements in household production diversity, maternal knowledge of child nutrition, and preschool children’s diets while also benefiting their younger siblings’ dietary diversity and linear growth. The study findings highlight that community-based ECD centers can provide a platform to change household behaviors related to food production and consumption, influencing decisions that may benefit all household members at different lifecycle stages. Moreover, evidence from this study indicates that the intervention had a protective effect during a period of high food insecurity, suggesting a role for these types of interventions within social protection portfolios. The intervention relies on community contributions and may provide a sustainable option for government scale-up.

## Authors’ contributions

This study was implemented in a partnership between IFPRI, Save the Children, and Chancellor College, U. of Malawi. We would like to acknowledge the different author contributions. At IFPRI: AG led the research design and development; AM provided inputs in the study design, questionnaire development, and enumerator training; MS for the data cleaning, processing, and analysis; HA and MR for inputs in the study design, data analysis, and manuscript preparation. At Save the Children: AT for supporting the enumerator training, translation, and survey management and coordination, without which the study would not have been possible; NR and HM for inputs in the study design and linkages with policy and program implementation. At Chancellor College, MK for inputs in the study design and the surveys. All authors read and approved the final manuscript.

## Acknowledgments

We are grateful to the Government of Malawi and acknowledge the inputs and feedback from Mr. McKnight Kalanda and Mr. Francis Chalamanda. We would like to thank the following experts for inputs and feedback on the study design: Christin McConnell (World Bank); Melissa Hidrobo (IFPRI), Peter Phiri (Save the Children) and Lexon Ndalama (Save the Children) for links with policy and program implementation.

We would like to thank Pamela Stedman-Edwards (IFPRI) for editorial inputs and feedback.

# References

1. Black RE, Victora C, Walker SP, Bhutta ZA, Christian P, De Onis M, Ezzati M, Grantham-McGregor S, Katz J, Matorell R, et al. Maternal and child undernutrition and overweight in low- and middle-income countries. *Lancet* 2013;382 (9890):427–51.
2. Development Initiatives. *Global Nutrition Report 2017: Nourishing the SDGs*. Bristol, UK: Development Initiatives;2017.
3. Bailey R L, West Jr. KP, Black RE. The epidemiology of global micronutrient deficiencies. *Ann Nutr Metab* 2015;66 (suppl 2):22–33
4. Masset E, Haddad L, Cornelius A, Isaza-Castro J. *A systematic review of agricultural interventions that aim to improve nutritional status of children.* London: EPPI-Centre, Social Science Research Unit, Institute of Education. University of London; 2011.
5. Ruel, MT, Alderman H, and the Maternal and Child Nutrition Study Group. Nutrition-sensitive interventions and programs: How can they help to accelerate progress in improving maternal and child nutrition? *Lancet* 2013;382 (9891): 536–51.
6. Ruel MT, Quisumbing AR, Balagamwala M. Nutrition-sensitive agriculture: What have we learned so far? *Glob Food Sec.* February 2018. doi:10.1016/J.GFS.2018.01.002.
7. Grantham-McGregor S M, Fernald L C H, Kagawa R MC., Walker S. Effects of integrated child development and nutrition interventions on child development and nutritional status. *Ann. N.Y. Acad. Sci.* 2014;1308: 11–32. doi:10.1111/nyas.12284.
8. Alderman H., Fernald L. The nexus between nutrition and early childhood development. *Annual Review of Nutrition* 2017;37:1, 447–476
9. Neuman MJ., McConnell C., Kholowa F. From early childhood development policy to sustainability: The fragility of community-based childcare services in Malawi. *International Journal of Early Childhood* 2014;46(1), 81–99. doi:10.1007/s13158-014-0101-1.
10. Gelli, A, Margolies A, Santacroce M, Sproule K, Theis S, Roschnik N, Katundu M. Improving child nutrition and development through community-based childcare centers in Malawi? The NEEP-IE study: study protocol for a randomized controlled trial. *Trials* 2017;18(1), 284. http://doi.org/10.1186/s13063-017-2003-7.
11. Olney DK, Rawat R, Ruel MT. Identifying potential programs and platforms to deliver multiple micronutrient interventions. *J Nutr.* 2012;142(1):178S–85S. doi: 10.3945/jn.110.137182.
12. Habicht JP, Victora CG, Vaughan JP. Evaluation designs for adequacy, plausibility and probability of public health programme performance and impact. *Int J Epidemiol.* 1999;28:10–8.
13. Malawi Demographic and Health Survey (DHS), 2015–16.
14. Government of Malawi. *The Malawi vulnerability assessment committee (MVAC) national food security forecast, April 2015 to March 2016.* Lilongwe, Malawi; 2015.
15. UNICEF. *Malawi humanitarian situation report.* Lilongwe, Malawi; 2015.
16. Government of Malawi. *The Malawi vulnerability assessment committee (MVAC) national food security forecast, April 2016 to March 2017.* Lilongwe, Malawi; 2016.
17. National Statistical Office. Malawi – Demographic and Health Survey 2016. Zomba, Malawi; 2016.
18. Gillespie S, Harris L, Kadiyala S. The agriculture-nutrition disconnect in India, what do we know? Discussion Paper 01187, International Food Policy Research Institute, Washington, DC; 2012.
19. Bundy DAP, Burbano C, Grosh M, Gelli A, Jukes M, Drake L. Rethinking school feeding: Social safety nets, child development, and the education sector. World Bank, 2009.
20. Steyn NP, Nel JH, Nantel G, Kennedy G, Labadarios D. Food variety and dietary diversity scores in children: are they good indicators of dietary adequacy? *Public Health Nutr* 2006;9:644–50.
21. Swindale A, Bilinsky P. *Household dietary diversity score (HDDS) for measurement of household food access: indicator guide* (v.2). Washington, DC: FHI 360/FANTA; 2006.
22. WHO. Indicators for assessing infant and young child feeding practices: conclusions of a consensus meeting held 6–8 November 2007 in Washington, DC.
23. Gibson RS, Ferguson EL. An interactive 24-hour recall for assessing the adequacy of iron and zinc intakes in developing countries. HarvestPlus Technical Monograph 8: Washington, DC and Cali, Colombia: International Food Policy Research Institute and International Center for Tropical Agriculture; 2008.
24. Willett W. *Nutritional epidemiology*. Oxford: Oxford University Press; 2013.
25. FAO (Food and Agriculture Organization of the United Nations) (2010). World food dietary assessment system, version 2.0. International network of food data systems of the food and agricultural organization of the United Nations. <http://www.fao.org/infoods/software_worldfood_>
26. United States Department of Agriculture. USDA table of retention factors, release 6. Beltsville, MD: United States Department of Agriculture; 2007.
27. Fiedler JL, Lividini K, Bermudez OI, Smitz MF. Household consumption and expenditures surveys (HCES): a primer for food and nutrition analysts in low- and middle-income countries. Food Nutr Bull 2012;33(3 Suppl):S170–84.
28. FAO and FHI 360. Minimum dietary diversity for women: a guide for measurement. Rome: FAO; 2016.
29. World Health Organization. *WHO child growth standards: length/ height-for-age, weight-for-age, weight-for-length, weight-for-height and body mass index-for-age: methods and development.* Geneva: World Health Organization; 2006.
30. FAO. Human energy requirements: Report of a Joint FAO/WHO/UNU Expert Consultation, Rome, 17-24 October 2001. Rome: FAO, 2004.
31. WHO. Protein and amino acid requirements in human nutrition: Report of a joint FAO/WHO/UNU expert consultation. Geneva: WHO, 2007.
32. Goldstein H. *Multilevel statistical models.* 3rd ed. London: Edward Arnold; 2003.
33. Hayes R., Moulton LH. *Cluster randomized trials.* Oxford, UK: Chapman & Hall/CRC Press; 2009.
34. Gelli A, Aberman N-L, Margolies A, Santacroce M, Baulch B, Chirwa E. Lean-season food transfers affect children’s diets and household food security: evidence from a quasi-experiment in Malawi. *J Nutr.* 2017: jn246652. doi:10.3945/jn.116.246652.
35. Penny ME, Creed-Kanashiro HM, Robert RC, Narro MR, Caulfield LE, Black RE. Effectiveness of an educational intervention delivered through the health services to improve nutrition in young children: a cluster-randomised controlled trial. *Lancet* 2005;365, 1863–1872.
36. Shi L, Zhang J, Wang Y, Caulfield L, Guyer B. Effectiveness of an educational intervention on complementary feeding practices and growth in rural China: A cluster randomised controlled trial. *Public Health Nutrition* 2010;13(4), 556–565. doi:10.1017/S1368980009991364.
37. Anderson ML. Multiple Inference and Gender Differences in the Effects of Early Intervention: A Reevaluation of the Abecedarian, Perry Preschool, and Early Training Projects. J Am Stat Assoc. 2008;103(484):1481-1495. doi:10.1198/016214508000000841.
38. Rawat R, Nguyen PH, Ali D, Saha K, Alayon S, Kim SS, Ruel MT, Menon P. Learning How Programs Achieve Their Impact: Embedding Theory-Driven Process Evaluation and Other Program Learning Mechanisms in Alive & Thrive. Food Nutr Bull. 2013;34(3_suppl2):S212-S225. doi:10.1177/15648265130343S207.

Table 1: Characteristics of the study population at baseline in treatment and control communities, Zomba district, Malawi^1^

| **Level** | **Variable** | **Treatment** | | ***n*** | **Control** | | ***n*** |
| --- | --- | --- | --- | --- | --- | --- | --- |
| Household | HH size, *n* | 5.32 (1.92) |  | 601 | 5.35 (1.68) |  | 598 |
|  | Children, *n*  0-36 mo | 0.50 (0.58) |  | 601 | 0.52 (0.58) |  | 598 |
|  | >36-72 mo | 1.13 (0.37) |  | 601 | 1.12 (0.35) |  | 598 |
|  | >6-14 y | 1.44 (1.21) |  | 601 | 1.47 (1.13 |  | 598 |
|  | Adults, *n*  >14-65 y | 2.25 (1.00) |  | 601 | 2.26 (0.97) |  | 598 |
|  | >65 y | 0.05 (0.24) |  | 601 | 0.04 (0.22) |  | 598 |
|  | Dependency ratio | 1.56 (0.96) |  | 601 | 1.6 (1.03 |  | 598 |
|  | HH head completed primary school, % | 32 |  | 601 | 38 |  | 598 |
|  | Household head's age, y | 36.8 (10.07) |  | 601 | 36.2 (10.3) |  | 598 |
|  | Polygamous households, % | 2 |  | 601 | 4 |  | 598 |
|  | Female-headed household, % | 27 |  | 601 | 29 |  | 598 |
|  | *Asset ownership, n* | |  |  |  |  |  |
|  | -Large livestock | 0.02 (0.24) |  | 601 | 0.05 (0.56) |  | 598 |
|  | -Small livestock | 0.87 (2.86) |  | 601 | 0.73 (1.9) |  | 598 |
|  | -Fowl (chickens) | 2.85 (5.42) |  | 601 | 2.30 (5.07) |  | 598 |
|  | -Farm equipment | 3.18 (2.57) |  | 601 | 3.07 (2.91) |  | 598 |
|  | -Small consumer durables | 14.49 (40.01) |  | 601 | 13.51 (14.8) |  | 598 |
|  | Total asset count | 23.57 (42.42) |  | 601 | 21.34 (18.9) |  | 598 |
|  | *Expenditures, MWK/d per capita* | |  |  |  |  |  |
|  | -Total^2^ | 252 (202) |  | 576 | 232 (172) |  | 563 |
|  | -Non-food^2^ | 61 (86) |  | 576 | 52 (66) |  | 563 |
|  | -Food^2^ | 191 (157) |  | 576 | 181 (143) |  | 563 |
|  |  |  |  |  |  |  |  |
| Mother | Completed primary school % | 19 |  | 962 | 21 |  | 956 |
|  | Age, y | 29.5 (7.51) |  | 859 | 29.9 (7.33) |  | 887 |
|  |  |  |  |  |  |  |  |
| Children | Girls, % | 50 |  | 962 | 52 |  | 956 |
|  | Stunting, 6-24 mo, % | 41 |  | 155 | 41 |  | 149 |
|  | Wasting, 6-24 mo, % | 1 |  | 158 | 3 |  | 150 |
|  | Underweight, 6-24 mo, % | 14 |  | 157 | 13 |  | 150 |
|  | Stunting, 36-72 mo, % | 40 |  | 615 | 39 |  | 601 |
|  | Wasting, 36-72 mo, % | 1 |  | 494 | 2 |  | 465 |
|  | Underweight, 36-72 mo, % | 17 |  | 517 | 17 |  | 494 |
|  | CBCC enrolment, % | 92 |  | 656 | 93 |  | 645 |
|  | CBCC attendance, last 5 days, % | 81 (27) |  | 576 | 77 (30) |  | 552 |
|  | Days CBCC open, last 5 days, *n* | 4.23 (1.42) |  | 606 | 4.38 (1.41) |  | 600 |
|  | Received meals, last 5 days, *n* | 0.24 (0.43) |  | 656 | 0.29 (0.45) |  | 645 |

**Notes**: ^1^All unadjusted baseline and endline values are means or percentages with standard deviations in parentheses. CBCC, Community-based childcare center; HH, household; MWK, Malawian Kwacha; pc, per capita. Asset count included 13 asset type categories where respondents indicated ownership and number of assets owned. ^2^Table excludes outliers for food consumption and total expenditure.

Table 2: Unadjusted mean household crop diversity and production of nutritious foods at baseline and after 12 mo in the intervention and control groups, and adjusted DID impact estimates in households living in treatment and control communities, in Zomba district, Malawi, NEEP-IE study^1^

|  | **Treatment** | | **Control** | | **DID** |  |
| --- | --- | --- | --- | --- | --- | --- |
| **Indicator** | **Baseline** | **Endline** | **Baseline** | **Endline** | **Impact** | **S.E.** |
| Crop production diversity score | 3.52 | 3.52 | 3.53 | 2.82 | 0.71*** | 0.10 |
| Crop production variety score | 6.62 | 7.87 | 6.54 | 5.67 | 2.14*** | 0.35 |
| Production of OFSP, kg | 1.55 | 5.62 | 1.47 | 1.05 | 4.32*** | 0.73 |
| Production of brown beans, kg | 3.71 | 0.70 | 2.86 | 0.70 | -0.90** | 0.44 |
| Production of pigeon peas, kg | 14.40 | 22.84 | 17.53 | 21.55 | 4.86** | 1.87 |
| Production of groundnuts, kg | 6.59 | 9.27 | 7.31 | 6.68 | 3.38** | 1.63 |
| Production of soya bean, kg | 0.37 | 1.73 | 0.16 | 0.08 | 1.45*** | 0.14 |
| Chickens owned, *n* | 2.84 | 3.40 | 2.30 | 1.71 | 1.16** | 0.45 |
| Egg production last 3 months, *n* | 4.44 | 5.46 | 3.83 | 1.23 | 3.44** | 1.21 |
| *n* | 542 |  | 580 |  |  |  |

***Notes****:* ^1^All unadjusted baseline and endline values are means. DID, difference in difference; S.E., standard error*. *P<0.10, **P<0.05, ***P<0.001.*

Table 3: CBCC meal provision, enrolment, and attendance, unadjusted mean at baseline and after 12 mo in the intervention and control groups, and adjusted DID impact estimates in children 36–72 mo at baseline living in treatment and control communities, Zomba district, Malawi, NEEP-IE study^1^

|  | **Treatment** | | **Control** | | **DID** |  |
| --- | --- | --- | --- | --- | --- | --- |
| **Indicator** | **Baseline** | **Endline** | **Baseline** | **Endline** | **Impact** | **S.E.** |
| Days center was open in last 7 days, *n* | 3.91 | 3.09 | 4.08 | 2.95 | 0.29** | 0.15 |
| Center provided meals, % | 23 | 46 | 29 | 40 | 10.6 pp** | 3.5 |
| Days meal provided in last 7 days, *n* | 0.75 | 1.47 | 0.92 | 1.13 | 0.51*** | 0.12 |
| Center enrolment, % | 92 | 64 | 93 | 60 | 4.6 pp* | 2.7 |
| Center attendance in last 7 days, % | 71 | 49 | 66 | 48 | -4.0 pp | 3.1 |
| *n* | 660 |  | 648 |  |  |  |

***Notes****:* ^1^All unadjusted baseline and endline values are means or percentages. DID, difference in difference; S.E., standard error. **P<0.10, **P<0.05, ***P<0.001.*

Table 4: Unadjusted mean daily dietary intake at home measured by quantitative 24-hr recall at baseline and after 12 mo in the intervention and control groups, and adjusted DID impact estimates, in children 36–72 mo at baseline living in treatment and control communities in Zomba district, Malawi, NEEP-IE study^1^

|  | **Treatment** | | **Control** | | **DID** | |
| --- | --- | --- | --- | --- | --- | --- |
| **Indicator** | **Baseline** | **Endline** | **Baseline** | **Endline** | **Impact** | **S.E.** |
| Food quantity, g | 566 | 846 | 595 | 720 | 153*** | 28.27 |
| Energy, kcal | 1273 | 1627 | 1321 | 1376 | 294*** | 50.30 |
| Protein, g | 40 | 54 | 42 | 48 | 8.12** | 2.64 |
| Iron, mg | 11 | 13 | 11 | 12 | 1.64** | 0.52 |
| Zinc, mg | 6 | 7 | 6 | 6 | 1.09** | 0.33 |
| Vitamin A, µg RAE | 449 | 930 | 600 | 1013 | 59.44 | 71.73 |
| Vitamin C, mg | 47 | 101 | 65 | 99 | 19.72** | 6.40 |
| Vitamin B-6, mg | 1.08 | 1.48 | 1.18 | 1.32 | 0.26*** | 0.06 |
| Vitamin B-12, µg | 0.53 | 0.92 | 0.66 | 0.73 | 0.31** | 0.16 |
| Individual dietary diversity score | 5.35 | 5.80 | 5.42 | 5.51 | 0.36*** | 0.09 |
| Individual food variety score | 7.22 | 7.60 | 7.04 | 6.86 | 0.55*** | 0.15 |
| *n* | 606 |  | 604 |  |  |  |

***Notes****:* ^1^All unadjusted baseline and endline values are means. DID, difference in difference; S.E., standard error; RAE, retinol activity equivalents*. *P<0.10, **P<0.05, ***P<0.001.*

Table 5: Unadjusted mean HAZ, WAZ, and WHZ, and prevalence of stunting, underweight, and wasting at baseline and after 12mo in the intervention and control groups, and adjusted DID estimates for these indicators, in children aged 36-72mo at baseline and 6-24mo at baseline living in treatment and control communities in Zomba district, Malawi, NEEP-IE study^1^

|  |  | **Treatment** | | **Control** | |  |  |
| --- | --- | --- | --- | --- | --- | --- | --- |
| **Age** | **Indicator** | **Baseline** | **Endline** | **Baseline** | **Endline** | **DID** | **S.E.** |
| 36-7 2mo | HAZ | -1.75 | -1.70 | -1.74 | -1.70 | 0.05 | 0.05 |
|  | Stunted (HAZ <-2 SDs), % | 40 | 36 | 39 | 36 | -1 pp | 2.6 |
|  | WAZ | -1.08 | -1.16 | -1.05 | -1.15 | 0.05 | 0.05 |
|  | Underweight (WAZ <-2 SDs), % | 17 | 34 | 17 | 32 | 2 pp | 0.03 |
|  | WHZ | 0.09 | -0.06 | 0.11 | 0.08 | -0.04 | 0.07 |
|  | Wasted (WHZ <-2 SDs), % | 1 | 1 | 1 | 2 | -1 pp | 0.01 |
|  | *N* | 631 |  | 617 |  |  |  |
| 6-24 mo | HAZ | -1.70 | -1.87 | -1.61 | -2.29 | 0.44** | 0.16 |
|  | Stunted (HAZ <-2 SDs), % | 41 | 45 | 42 | 63 | -17 pp ** | 5.8 |
|  | WAZ | -0.68 | -1.05 | -0.73 | -1.18 | -0.02 | 0.14 |
|  | Underweight (WAZ <-2 SDs), % | 14 | 16 | 13 | 22 | -5 pp | 0.04 |
|  | WHZ | 0.12 | 0.04 | 0.09 | 0.09 | -0.13 | 0.15 |
|  | Wasted (WHZ <-2 SDs), % | 1 | 2 | 3 | 1 | 4pp | 0.02 |
|  | *n* | 155 |  | 149 |  |  |  |

**Notes**: ^1^All unadjusted baseline and endline values are means or percentages. DID, difference in difference; S.E., standard error; HAZ, height-for-age z-score; WAZ, weight-for-age z-score; WHZ, weight-for-height z-score. **P<0.10, **P<0.05, ***P<0.001.*

Figure 1: Schematic view of the program impact pathways for the integrated agriculture and nutrition intervention in the NEEP-IE study.

Figure 2: Schematic view of the randomization process and trial profile. ECD, Early Childhood Development.

Figure 3: Proportion of RDA provided by meals in CBBCs (CBCC n=25) the day prior to the survey, Zomba district, Malawi, NEEP-IE study. CBCC, Community Based Childcare Centers; RDA, Recommended Daily Allowance.

Figure 4: Baseline, midline, and endline unadjusted mean HAZ score (with 95% C.I.) by study group in children 6-24mo at baseline, Zomba district, Malawi, NEEP-IE study. Baseline n=304, midline n=244, endline n=244.
