# Supplementary material for: Using a Community-Based Early Childhood Development Center as a Platform to Promote Production and Consumption Diversity Increases Children's Dietary Intake and Reduces Stunting in Malawi: A Cluster-Randomized Trial
Source: J Nutr. 2018 Sep 10;148(10):1587–97. doi: 10.1093/jn/nxy148 (PMC6168702; doi:10.1093/jn/nxy148)
Supplement: Supplemental Files [file nxy148_supplemental_files.zip › Gelli_NEEPIE_JoN_12062018_osm.docx]

**Supplementary Data**

Supplemental Table 1: Unadjusted means and difference in means at baseline between attrited and non-attrited children for key outcomes, Zomba district, Malawi, NEEP-IE study^1^

|  |  | **Non-attrited** | | **Attrited** | |  |
| --- | --- | --- | --- | --- | --- | --- |
| **Age group** | **Variable** | ***n*** | **Mean** | ***n*** | **Mean** | **Difference** |
| 6-24 mo | HAZ | 243 | -1.63 | 71 | -1.70 | 0.07 |
| 36-72 mo | HAZ | 1252 | -1.74 | 394 | -1.70 | -0.04 |
|  | Food quantity, g | 1052 | 572.38 | 134 | 589.26 | -16.88 |
|  | Energy, kcal | 1052 | 1269.93 | 134 | 1327.18 | -57.26 |
|  | Protein, g | 1052 | 40.49 | 134 | 41.79 | -1.30 |
|  | Iron, mg | 1052 | 10.74 | 134 | 11.41 | -0.67 |
|  | Zinc, mg | 1052 | 5.86 | 134 | 5.96 | -0.10 |
|  | Vitamin A, µg RAE | 1052 | 524.78 | 134 | 523.67 | 1.11 |
|  | Vitamin C | 1052 | 55.28 | 134 | 57.67 | -2.40 |
|  | Vitamin B-6 | 1052 | 1.11 | 134 | 1.13 | -0.02 |
|  | Vitamin B-12 | 1052 | 0.57 | 134 | 0.57 | 0.01 |
|  | DDS | 1052 | 7.16 | 134 | 7.03 | 0.13 |
|  | FVS | 1052 | 5.39 | 134 | 5.37 | 0.02 |

*^1^* All unadjusted baseline and endline values are means. HAZ, height-for-age z-score; DDS, dietary diversity score; FVS, food variety score; RAE, retinol activity equivalents. **P<0.10, **P<0.05, ***P<0.001.*

Supplementary Data

Supplemental Table 2: Unadjusted mean caregiver knowledge of IYCF practices, and adjusted DID impact estimates, Zomba district, Malawi, NEEP-IE study*^1^*

(Percent of caregivers who provided the following responses when asked “What do you know about the needs of children 0–6 months regarding feeding?”)

|  | **NEEP** | | **Control** | | **DID** | |  |
| --- | --- | --- | --- | --- | --- | --- | --- |
| **IYCF Knowledge** | **Baseline** | **Endline** | **Baseline** | **Endline** | **Impact (pp)** | **S.E.** | |
| Initiate breastfeeding within 1 hour of delivery | 14% | 25% | 25% | 28% | 7.9** | 3.4 | |
| No water for first 6 months | 44% | 55% | 46% | 44% | 13.5** | 4.0 | |
| Exclusive breastfeeding for 6 months | 85% | 84% | 71% | 64% | 5.0 | 3.4 | |
| Breastfeed on demand | 5% | 9% | 12% | 9% | 7.4** | 2.3 | |
| Give baby colostrum | 7% | 16% | 9% | 7% | 10.6*** | 2.4 | |
| Allow baby to suckle long at 1 breast | 3% | 6% | 6% | 5% | 3.8** | 1.8 | |
| Introduce complementary foods at 6 months | 82% | 78% | 74% | 69% | 1.0 | 3.5 | |
| Feed child a range of foods from all food groups | 58% | 52% | 50% | 51% | -6.7 | 4.1 | |
| Give frequent small meals | 13% | 21% | 18% | 18% | 7.5** | 3.1 | |
| Cook separately for baby without harsh spices | 7% | 16% | 6% | 7% | 7.9** | 2.3 | |
| Be patient & encourage child to eat during feeding time | 6% | 11% | 5% | 6% | 5.0** | 2.1 | |
| Continue breastfeeding until child is 2 years | 37% | 35% | 35% | 25% | 6.7* | 3.8 | |
| *n* | 608 |  | 602 |  |  |  | |

*^1^* All unadjusted baseline and endline values are mean percentages. IYCF, infant and young child feeding. DID, difference in difference; S.E., standard error. **P<0.10, **P<0.05, ***P<0.001.*

Supplementary Data

Supplemental Table 3: Unadjusted mean caregiver knowledge of food groups and adjusted DID impact estimates, Zomba district, Malawi, NEEP-IE study*^1^*

(Percent of caregivers who provided the following responses when asked “Name the food groups you know?”)

|  | **NEEP** | | **Control** | | **DID** | |
| --- | --- | --- | --- | --- | --- | --- |
| **Knowledge of food groups** | **Baseline** | **Endline** | **Baseline** | **Endline** | **Impact** | **S.E.** |
| Animal products | 58% | 66% | 58% | 55% | 9.6 pp ** | 3.7 |
| Staples / carbohydrates | 57% | 66% | 49% | 43% | 15.1 pp *** | 3.7 |
| Vegetables | 61% | 69% | 60% | 57% | 10.7 pp ** | 3.7 |
| Fruits | 53% | 62% | 55% | 45% | 18.4 pp *** | 3.7 |
| Added fats and oils | 40% | 49% | 28% | 25% | 11.7 pp *** | 3.3 |
| Pulses | 49% | 57% | 35% | 33% | 11.1 pp ** | 3.8 |
| Food group knowledge score | 2.59 | 3.68 | 2.85 | 3.18 | 0.77*** | 0.16 |
| *n* | 594 |  | 594 |  |  |  |

*^1^* All unadjusted baseline and endline values are means. DID, difference in difference; S.E., standard error. **P<0.10, **P<0.05, ***P<0.001.*

Supplementary Data

Supplemental Table 4: Unadjusted mean caregiver sources of knowledge and adjusted DID impact estimates, Zomba district, Malawi, NEEP-IE study*^1^*

(Percent of caregivers who provided the following responses when asked “Who/where did you receive this information concerning 0-5-year-old children from?”)

|  | **Treatment** | | **Control** | | **DID** |  |
| --- | --- | --- | --- | --- | --- | --- |
| **Knowledge sources** | **Baseline** | **Endline** | **Baseline** | **Endline** | **Impact (pp)** | **S.E.** |
| Health worker/Health facility | 86% | 92% | 90% | 94% | 2 | 0.02 |
| Teacher/preschool staff | 2% | 5% | 2% | 3% | 2 | 0.01 |
| Community volunteer | 2% | 6% | 3% | 8% | -19 | 0.02 |
| Child | 0% | 1% | 0% | 0% | 1 | 0.01 |
| Friend | 4% | 4% | 2% | 5% | -4** | 0.02 |
| Radio/Television | 5% | 4% | 3% | 2% | -1 | 0.01 |
| NGO Save the Children | 2% | 28% | 4% | 14% | 16*** | 0.02 |
| Another NGO (specify) | 1% | 1% | 1% | 1% | 0 | 0.01 |
| Church/Mosque | 0% | 2% | 0% | 2% | 0 | 0.01 |
| *n* | 608 |  | 602 |  |  |  |

*^1^* All unadjusted baseline and endline values are means. DID, difference in difference; S.E., standard error. **P<0.10, **P<0.05, ***P<0.001.*

Supplementary Data

Supplemental Table 5: Unadjusted mean HAZ, WAZ, and WHZ, and prevalence of stunting, underweight, and wasting at baseline and after 12 mo, and adjusted DID estimates for these indicators in children aged 6–24 mo at baseline in the intervention and control groups, full sample and in full cohort subsample in Zomba district, Malawi, NEEP-IE study^1^

|  | **Full sample (6-24mo)** | | | | **Cohort only sample (6-24mo)** | | | |
| --- | --- | --- | --- | --- | --- | --- | --- | --- |
| **Indicator** | **Treatment** | **Control** | **DID (t1, t2)** | **DID (t2)** | **Treatment** | **Control** | **DID (t1, t2)** | **DID (t2)** |
| HAZ, *n* | 155 | 149 |  |  | 110 | 98 |  |  |
| Baseline | -1.7 ±1.43 | -1.61 ±1.42 |  |  | -1.56 ±1.55 | -1.62 ±1.26 |  |  |
| Midline (t1) | -2.26 ±1.75 | -2.38 ±1.27 | 0.26 ±0.18 |  | -2.23 ±1.79 | -2.3 ±1.27 | 0.01 ±0.196 |  |
| Endline (t2) | -1.87 ±1.44 | -2.29 ±1.2 | 0.47** ±0.18 | 0.44** ±0.16 | -1.84 ±1.43 | -2.26 ±1.17 | 0.33* ±0.196 | 0.37** ±0.17 |
| Stunted (HAZ<-2) |  |  |  |  |  |  |  |  |
| Baseline, % | 41 | 42 |  |  | 39 | 40 |  |  |
| Midline (t1), % | 55 | 57 | -1.7 ±6.1 |  | 53 | 56 | -0.4 ±6.9 |  |
| Endline (t2), % | 45 | 63 | -17** ±6.1 | -17** ±5.8 | 43 | 63 | -17** ±6.9 | -18** ±7 |

^1^All unadjusted baseline and endline values are means. DID, difference in difference; HAZ, height-for-age z-score; WAZ, weight-for-age z-score; WHZ, weight-for-height z-score. **P<0.10, **P<0.05, ***P<0.001.*
